# Fabrication of a Novel Optical Glucose Biosensor Using Copper(II) Neocuproine as a Chromogenic Oxidant and Glucose Dehydrogenase-Immobilized Magnetite Nanoparticles

**DOI:** 10.1021/acsomega.3c07181

**Published:** 2023-12-02

**Authors:** Selen Ayaz, Ayşem Üzer, Yusuf Dilgin, M. Reşat Apak

**Affiliations:** † Faculty of Science, Department of Chemistry, Canakkale Onsekiz Mart University, Canakkale 17100, Turkey; ‡ Faculty of Engineering, Department of Chemistry, İstanbul University-Cerrahpaşa, İstanbul-Avcılar 34320, Turkey

## Abstract

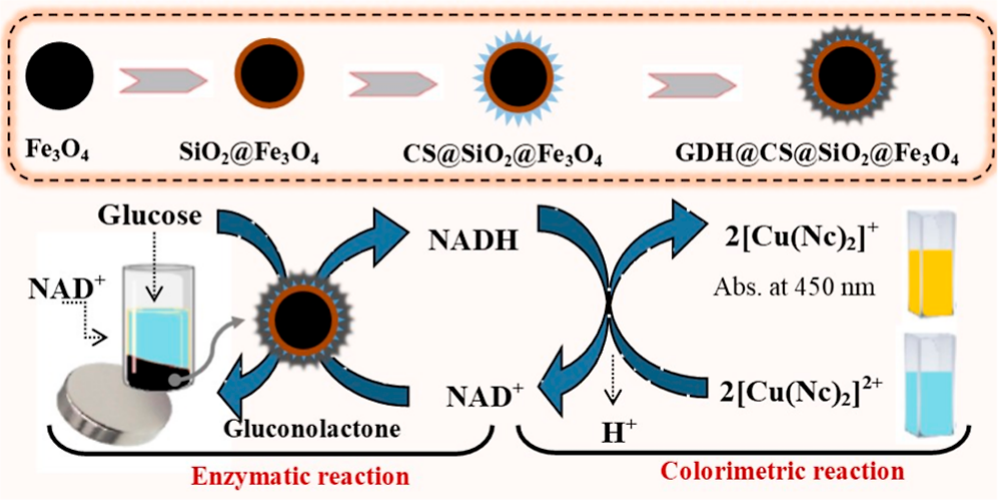

This study describes a novel optical glucose biosensor based on a colorimetric reaction between reduced nicotinamide adenine dinucleotide (NADH) and a copper(II) neocuproine complex ([Cu(Nc)_2_]^2+^) as a chromogenic oxidant. An enzymatic reaction takes place between glucose and glucose dehydrogenase (GDH)–chitosan (CS) immobilized on silanized magnetite nanoparticles (CS@SiO_2_@Fe_3_O_4_) in the presence of coenzyme NAD^+^. The oxidation of glucose to gluconolactone via the immobilized enzyme is coupled with the reduction of NAD^+^ to NADH at the same time. After the separation of GDH-immobilized SiO_2_@Fe_3_O_4_ with a magnet, the enzymatically produced NADH chemically reduces the chromogenic oxidant cupric neocuproine to the cuprous chelate. Thus, the glucose biosensor is fabricated based on the measurement of the absorbance of the formed yellow-orange complex ([Cu(Nc)_2_]^+^) at 450 nm. The obtained results show that the colorimetric biosensor has a wide linear response range for glucose, between 1.0 and 150.0 μM under optimized conditions. The limit of detection and limit of quantification were found to be 0.31 and 1.02 μM, respectively. The selectivity properties of the fabricated biosensor were tested with various interfering species. This biosensor was applied to various samples, and the obtained results suggest that the fabricated optical biosensor can be successfully used for the selective and sensitive determination of glucose in real samples.

## 1 Introduction

Diabetes is a chronic disease that is becoming increasingly prevalent worldwide, threatening human health, causing various diseases, and even leading to death if no precautions are taken.^1^ Therefore, it is very important to monitor blood glucose levels in terms of human health, especially for diabetic and prediabetic patients. This vital importance of glucose in human health has made it one of the leading molecules for the design of both enzymatic and nonenzymatic biosensors over the last few decades.^2−8^ In this context, electrochemical methods have attracted a great deal of attention from most researchers involved in biosensor studies.^2−5^ Moreover, optical glucose biosensor studies based on absorbance, fluorescence, chemiluminescence, surface plasmon resonance, and interferometry measurements have maintained their place on this great sensing platform.^6−8^ Among the optical transducers, colorimetric assays based on absorbance measurement have recently gained great attention due to their several advantages, such as simplicity, low cost, high sensitivity, and naked eye perception of color signals without needing sophisticated equipment.^6,9^

One of the procedures in the fabrication of the glucose biosensor is the use of the nicotinamide adenine dinucleotide [NAD(P)^+^/NAD(P)H] redox pair-dependent dehydrogenase enzymes.^9−11^ NADH and its phosphate form (NADPH) are biological molecules that have an important role as cofactors in the realization of various enzymatic reactions required for cellular and metabolic activities. Therefore, the determination of NADH is very important in the elucidation of metabolic mechanisms and the diagnosis and treatment of related diseases.^12^ In addition, the NAD^+^/NADH redox couple has been widely used in the design of dehydrogenase-based electrochemical or optical biosensors due to it acting as a cofactor for more than 300 dehydrogenase enzymes.^9−14^ Thus, the determination of many substrates has been reported based on the monitoring of the signal of the enzymatically produced NADH. NAD(P)H determination or NADH-dependent direct or fiber optic biosensor design using specific dehydrogenase enzymes for various substrates such as phenylalanine, sorbitol, and alcohol was carried out by monitoring its absorbance at 340 nm or the measurement of fluorescence intensity at 450 or 490 nm after excitation at 340 nm.^15−20^ However, the sensitivity and selectivity of this direct measurement are not sufficient at low NADH concentrations.

To overcome this problem, considerable attention has been paid to enzymatic biosensors based on various types of colorimetric reactions. The reason is that simple, cheap, highly selective, and sensitive analyses can be performed by using the specificity properties of enzymes. Moreover, the change in optical properties of chromogenic compounds through their reaction with enzymatically produced products is another advantage of enzyme-based colorimetric biosensors. One of the approaches to colorimetric sensors and biosensors is the use of changes in the colorimetric properties of plasmonic nanoparticles such as Au NPs, Au@Cu NPs, and Ag NPs after their reaction with NAD(P)H.^9,21−26^ Direct NAD(P)H, glucose-6-phosphate dehydrogenase (G-6-PDH) deficiency, glucose, and lactate determinations were performed by using this approach. In another approach, biosensors based on the dehydrogenase enzyme have been developed by monitoring the absorbance of the blue-colored oxidized form of 3,3′,5,5′-tetramethylbenzidine (TMB_ox_) at 652 nm. TMB_ox_ was produced by catalyzing the oxidation of its colorless reduced form (TMB_red_) with peroxidase-mimic nanoparticles such as Ag nanostars-metal organic framework,^12^ CuWO_4_ NPs,^27^ and MnO_2_ NPs,^28^ in the presence of H_2_O_2_.

Another alternative approach is to use a chromogenic molecule or probe that reacts directly with NAD(P)H. At the end of the colorimetric reaction, either the color of the chromogen changes to another color or the colorless compound turns into a colored one. Thus, the biosensor design is carried out by measuring the absorbance (in the visible region) or fluorescence intensity. In this context, several water-soluble tetrazolium salts (WSTs or MTTs) have been synthesized for the determination of NADH and the fabrication of dehydrogenase-based biosensors.^29−35^ Generally, yellow WST dyes are converted to formazan forms with different colors (generally purple), while NADH is oxidized to NAD^+^ after the colorimetric reaction. Apart from these chromogenic reagents, a well-known and frequently used oxidizing reagent is the bis(neocuproine) copper(II) complex, known as the CUPRAC reagent, developed by Apak et al. in 2004.^36^ With this reagent, ascorbic acid,^37^ hydrogen peroxide scavenging,^38^ catalase, and xanthine oxidase activities in the presence of phenolic compounds^39,40^ were determined besides the spectrophotometric total antioxidant capacity (TAC) determination. Moreover, the CUPRAC reagent has recently been used for the first time in enzymatic biosensors using glucose oxidase, uricase, and choline oxidase enzymes,^41^ xanthine oxidase,^42^ and an enzymatic organophosphate pesticide biosensor with the use of acetylcholine esterase undergoing pesticide inhibition.^43^ However, the interaction of this useful chromogenic oxidizing reagent with NADH, leading to the fabrication of biosensors based on dehydrogenase enzymes, has not yet been performed.

This study describes the fabrication of a novel optical glucose biosensor using glucose dehydrogenase (GDH) immobilized onto silanized magnetite nanoparticles (MNPs) and the CUPRAC reagent. Although GDH-based optical biosensors were reported using different types of chromogenic oxidants, as mentioned above, the integration of the CUPRAC method into biosensors based on dehydrogenase enzymes has not been reported yet. As opposed to the indefinite stoichiometry of redox-active dyes and chromogens such as TMB and *o*-dianisidine used to detect H_2_O_2_ generated from glucose oxidase-catalyzed oxidation of glucose, the cupric-neocuproine reagent has a clear stoichiometry of being one electron reduced to the cuprous chelate. Thus, the novelty of this study is the first-time investigation of the colorimetric reaction between the CUPRAC reagent and NADH and the integration of a commonly used chromogenic oxidizing reagent, cupric neocuproine chelate, into a dehydrogenase-based biosensor. Based on the CUPRAC reagent and the immobilized GDH enzyme, a highly selective and sensitive optical determination of glucose is developed. Furthermore, the fabricated biosensor has been successfully applied to glucose determination in samples, such as real human blood, artificial blood, and some beverages.

## 2 Experimental Section

### 2.1 Spectrophotometric Measurements

First, spectrophotometric measurements were performed based on a colorimetric reaction between [Cu(Nc)_2_]^2+^ and NADH. For this, an adequate volume of standard NADH solution with a known concentration was diluted to 2.5 mL with water after adding 0.75 mL of 2.25 mM neocuproine (Nc) prepared in ethanol, 0.50 mL of 2.0 mM CuCl_2_, and 0.75 mL of 1.0 M NH_4_CH_3_COO. By using this experimental procedure, both the optimization (pH, colorimetric reaction time, and temperature) and analytical performance studies were carried out by recording the absorbance (at 450 nm) or spectrum (between 340 and 800 nm) of the yellow-colored [Cu(Nc)_2_]^+^ formed.

In the second step, spectrophotometric measurements were performed on the enzymatic glucose biosensor. For this, 15 mg of GDH@CS@SiO_2_@Fe_3_O_4_ NPs was added to 0.75 mL of 1.0 M NH_4_CH_3_COO, which included both 10.0 mM NAD^+^ and a known concentration of glucose. After incubation for 30 min for the completion of the enzymatic reaction, MNPs were separated with a magnet and washed with pH 5.0 phosphate buffer solution (PBS) several times before being used again. After separation, 0.75 mL of 2.25 mM Nc, 0.50 mL of 2.0 mM CuCl_2_, and the required volume of water to make up the final volume of 2.5 mL were added to the remaining solution. Similar studies were also carried out using the enzyme directly in solution without immobilization (free enzyme). Parameter optimization (pH, temperature, enzymatic reaction period, amount of enzyme, amount of MNPs, etc.) and analytical performance studies for glucose were completed by recording the spectrum and absorbance of the yellow complex formed between the CUPRAC reagent and enzymatically produced NADH.

### 2.2 Real Sample Analysis

Four different types of real samples (beverages, human blood serum, commercial dextrose serum, and glucose tolerance test drink) and one artificial blood sample were used to assess the applicability of the fabricated glucose biosensor. Human blood plasma samples were collected from three volunteers at Medical Park Hospital, Çanakkale, Turkey. Commercial dextrose solutions, including 5% dextrose monohydrate (calculated concentration: 252.5 mM glucose), beverages (soda, ice tea, and fruit juice), and an oral glucose tolerance test drink, including 75.0 g of dextrose monohydrate per 250 mL (calculated concentration: 1515.2 mM glucose), were purchased from a local market or drugstore. The experiments using human serum samples were approved and conducted according to the guidelines of the Çanakkale Onsekiz Mart University (Turkey) Ethics Committee (no. 2011-KAEK-27/2021-E.210002529). An artificial blood serum sample was prepared according to our previously reported study.^44^ All samples were diluted at a known ratio with pH 7.0 PBS, including 10 mM NAD^+^. To detect glucose, the absorbance of samples before and after the addition of a known standard glucose solution was measured at 450 nm after enzymatic and colorimetric reactions were completed. All samples were also analyzed in Medical Park Hospital, Çanakkale, Turkey, by a validated enzymatic method based on the spectrophotometric determination of enzymatically produced NADPH using hexokinase and G6-PDH dye at 340 and 700 nm.^45^

## 3 Results and Discussion

### 3.1 Characterization of Synthesized and Enzyme-Immobilized Fe_3_O_4_ NPs

**Figure 1:**
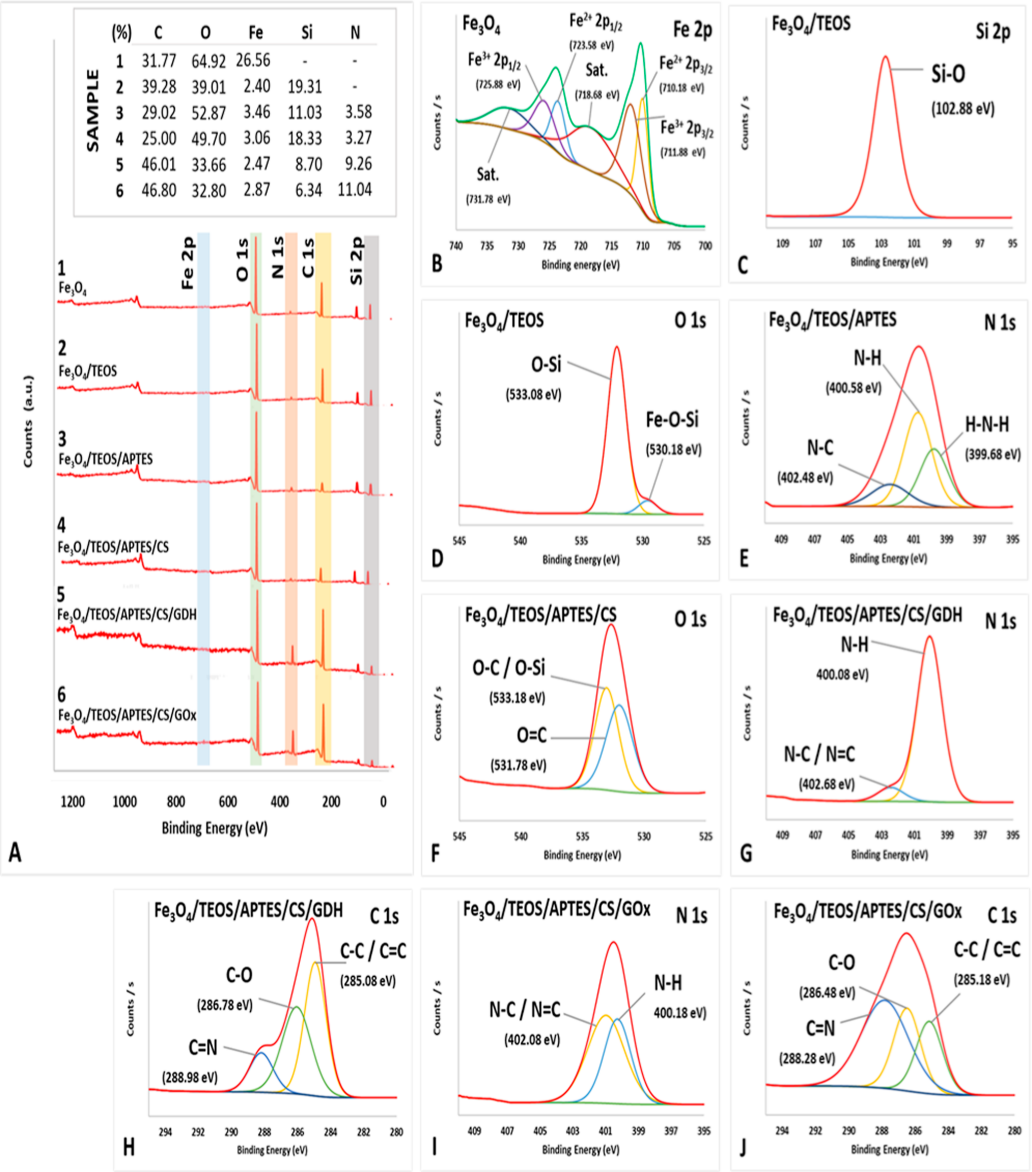
(A) XPS survey spectra and atomic (%) contents of 1, 2, 3, 4, 5, and 6. (B) Fe 2p XPS spectrum of 1. (C) Si 2p and (D) O 1s XPS spectra of 2. (E) N 1s XPS spectrum of 3. (F) O 1s XPS spectrum of 4. (G) N 1s and (H) C 1s XPS spectra of 5. (I) N 1s and (J) C 1s XPS spectra of 6 (1: Fe_3_O_4_ NPs, 2: Fe_3_O_4_/TEOS, 3: Fe_3_O_4_/TEOS/APTES, 4: Fe_3_O_4_/TEOS/APTES/CS, 5: Fe_3_O_4_/TEOS/APTES/CS/GAL, and 6: Fe_3_O_4_/TEOS/APTES/CS/GDH).

To characterize MNPs in all steps of synthesis, silanization, and enzyme immobilization, their Fourier transform infrared (FTIR), energy-dispersive X-ray (EDX), XRD, and X-ray photoelectron spectroscopy (XPS) spectra and transmission electron microscopy (TEM) and scanning electron microscopy (SEM) images were recorded. Here, only XPS spectra were presented in Figure 1, and a detailed discussion of other characterization results was given in the Supporting Information file (Figures S1–S7).

MNPs (1; Fe_3_O_4_ NPs), tetraethyl orthosilicate (TEOS)-modified MNPs (2; Fe_3_O_4_/TEOS), 3-aminopropyltriethoxysilane (APTES)-modified Fe_3_O_4_/TEOS (3; Fe_3_O_4_/TEOS/APTES), chitosan (CS)-modified Fe_3_O_4_/TEOS/APTES (4; Fe_3_O_4_/TEOS/APTES/CS), glutaraldehyde (GAL)-modified Fe_3_O_4_/TEOS/APTES/CS (5: Fe_3_O_4_/TEOS/APTES/CS/GAL), and GDH enzyme-immobilized Fe_3_O_4_/TEOS/APTES/CS (6: Fe_3_O_4_/TEOS/APTES/CS/GDH) formed at the end of the applied procedure were analyzed.

Figure 1A shows the XPS survey spectra and percentage atomic contents of bare and modified MNPs with different modification steps. The observed five atoms for the bare and magnetite nanoparticles are Fe, O, C, N, and Si. When the high-resolution Fe 2p spectrum is examined, characteristic 2p orbital peaks of Fe^2+^ and Fe^3+^ are observed at 710.18 and 725.88 eV and at 711.88 and 731.78 eV (Figure 1B), respectively. These peaks confirm the two different oxidation states of Fe in MNPs. The peak of the Si–O (102.88 eV) bond observed in Figure 1C and the peak of the Fe–O–Si (530.18 eV) bond observed in Figure 1D prove that TEOS binds to MNPs. However, the characteristic three peaks of N 1s observed at 399.68, 400.58, and 402.48 eV (Figure 1E) can be attributed to H–N–H, N–H, and N–C bonds, respectively. These bonds confirm that APTES is successfully modified on Fe_3_O_4_/TEOS. For Fe_3_O_4_/TEOS/APTES/CS prepared with CS modification, O–C/O–Si bond peaks at 533.18 eV and O=C bond peaks at 531.78 eV are seen in the high-resolution XPS spectrum of O 1s (Figure 1F). These peaks indicate the modification of CS on the nanoparticle surface. High-resolution O 1s and C 1s spectra taken to prove the presence of GAL on the nanoparticle surface are shown in Figure 1G,H, respectively. A comparison of Figure 1F and 1G reveals that while the intensity of the O–C/O–Si (533.08 eV) bond peak in Fe_3_O_4_/TEOS/APTES/CS/GAL decreases, the intensity of the O=C (531.68 eV) bond peak increases (Figure 1G). This confirms that a new layer is formed on the nanoparticle surface and therefore the O–Si band intensity decreases. The increase in the intensity of the O=C peak can be attributed to O=C bonds in the GAL structure. When the C 1s spectrum of Fe_3_O_4_/TEOS/APTES/CS/GAL is examined, C–C bond peaks are observed at 285.18 eV, C=O at 286.48 eV, and C–N/C=N bond peaks at 288.28 eV (Figure 1H). The C–C and C=O peaks support the successful binding of GAL to the nanoparticle surface. The appearance of the C–N/C=N bond peak is due to the CS layer existing on the nanoparticle surface before GAL. Finally, Figure 1I,J shows high-resolution N 1s and C 1s spectra taken to show that GDH is immobilized on Fe_3_O_4_/TEOS/APTES/CS. The N peak of the N–C/N=C bonds is observed at 402.68 eV, and the N peak of the N–H bond is observed at 400.08 eV (Figure 1I). C–C/C=C, C–O, and C=N peaks seen in the C 1s spectrum at 285.18, 286.48, and 288.28 eV, respectively, confirm the presence of GDH on the nanoparticle surface (Figure 1J).

### 3.2 Spectrophotometric Measurements Based on the Colorimetric Reaction between NADH and [Cu(Nc)_2_]^2+^

In the literature, optical sensors or biosensors based on the direct or indirect colorimetric reactions between various types of molecules (ascorbic acid, glucose, choline, hypoxanthine, phenolic compounds, and H_2_O_2_) and the CUPRAC reagent have been developed.^36−38,41,42^ However, our literature search shows that no studies based on its reaction with NADH have been reported. Thus, spectrophotometric measurements based on NADH and CUPRAC reagents were performed for the first time in this study. First, certain parameters such as pH, colorimetric reaction time, and concentration of both Cu(II) and Nc were optimized through the interaction of NADH at two different concentrations (50 and 125 μM) with [Cu(Nc)_2_]^2+^. Absorbance measurements at 450 nm after completion of the colorimetric reaction show that pH within a reasonable range has no effect on the absorbance of [Cu(Nc)_2_]^+^, which forms proportionally with NADH. The experiments were continued at conditions near physiological pH 7.0 using 1.0 M NH_4_CH_3_COO. In addition, 750 μL of 2.25 mM Nc prepared in ethanol, 500 μL of 2.0 mM CuCl_2_, 750 μL of pH 7.0 1.0 M NH_4_COOH, (500 – *x*) μL water [*x* is the volume (μL) of standard NADH], 2.5 mL of total volume, and 30 min of the colorimetric reaction were determined as optimal reaction conditions.

**Figure 2:**
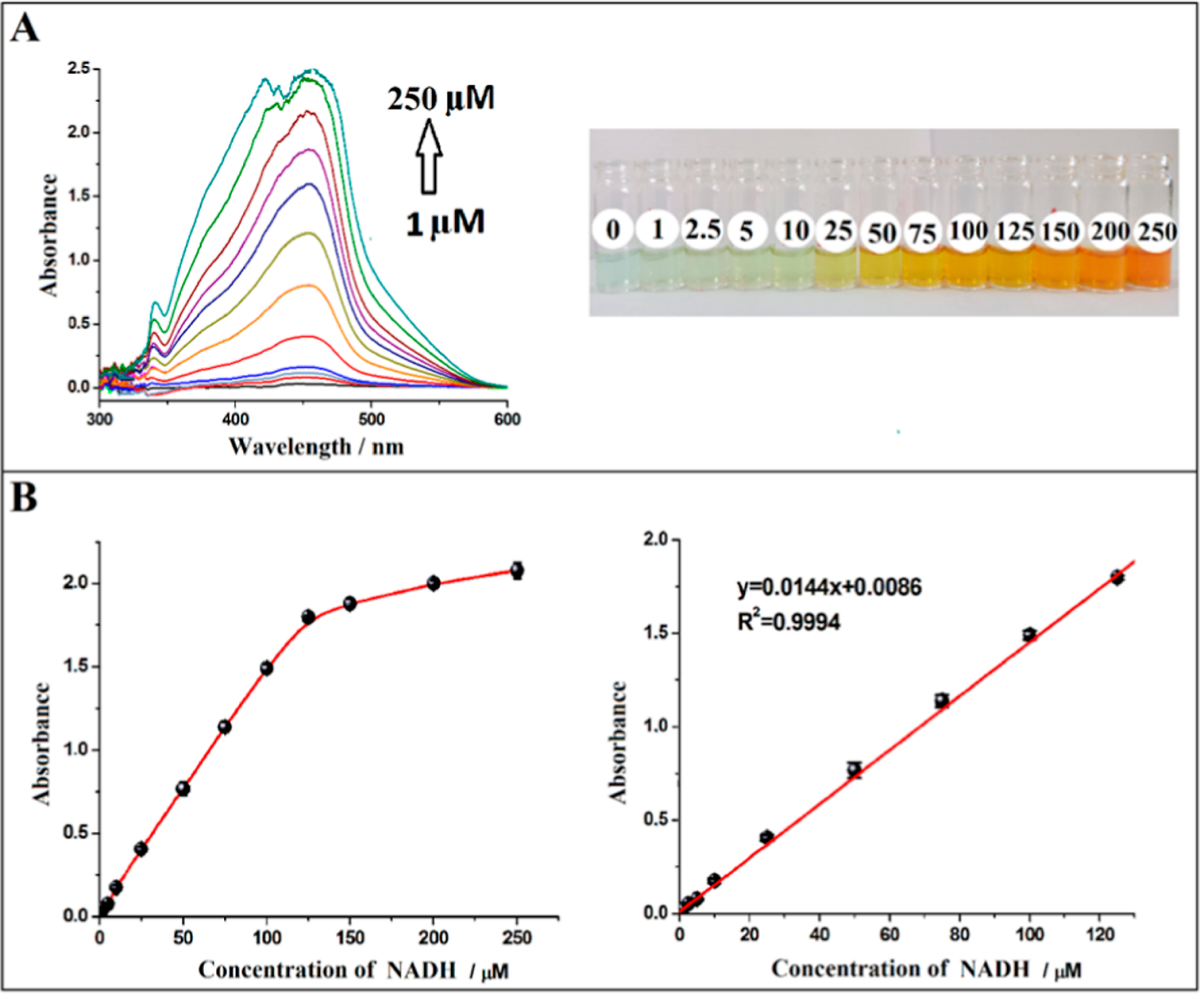
(A) Spectra and photographs of [Cu(Nc)_2_]^+^ formed as a result of the reaction between [Cu(Nc)_2_]^2+^ and NADH at different concentrations; and (B) curves obtained from nonlinear and linear regions based on absorbance values recorded at 450 nm for NADH at different concentrations.

The absorbance values corresponding to increasing NADH concentrations in the range of 1.0 to 200.0 μM were measured at 450 nm after the colorimetric reaction was completed under optimal conditions. In addition, the spectrum and photography of the yellow-colored complex of [Cu(Nc)_2_]^+^ formed proportionally to the NADH concentration were recorded and presented in Figure 2A. As can be seen from these figures, the intensity of the color increased as the NADH concentration increased, and the absorption peaks at 450 nm also increased depending on the increased NADH concentration. Figure 2B shows the curves of the absorbance versus concentration in the nonlinear and linear regions, respectively. The linear dynamic range was found to be between 1.0 and 125.0 μM with the equation *A* = 0.0144*C* (μM) + 0.0086. The wideness of the linear range originates from the definite stoichiometry of the CUPRAC reaction, where a single chromophore (cuprous neocuproine) absorbs light at 450 nm, minimizing chemical deviations from Beer’s law. The slope corresponding to the molar absorption coefficient (ε) of NADH in the CUPRAC method was estimated as 1.44 × 10^4^ L mol^–1^ cm^–1^. As the molar absorptivity of cuprous-neocuproine chelate is known, NADH acts as a 2-e^–^ reductant toward the cupric-neocuproine reagent, which is consistent with the physiological role of reduced NADH supplying two electrons to the mitochondrial electron transport.^46^ The limit of detection (LOD) and limit of quantification (LOQ) were calculated as 0.48 and 1.59 μM, respectively. In this context, the absorbance values of the blank, which does not include NADH, were measured at 450 nm (*n* = 5) and the standard deviation of the absorbance of the blank (*s*_b_) was calculated. Then LOD and LOQ values were found using the equations: LOD = 3*s*_b_/*m* and LOQ = 10*s*_b_/*m*, where *m* is the slope of the calibration line. To evaluate repeatability, the absorbance for two different concentrations (10 and 100 μM) of NADH was measured three times, and the RSD values were found to be 4.8 and 2.6% for 10 and 100 μM, respectively. These values show that proposed NADH sensing has very good repeatability.

### 3.3 Studies on Optical Glucose Biosensors Using GDH@CS@SiO_2_@Fe_3_O_4_ NPs

GDH-based glucose biosensor studies were performed in solution media using the free enzyme (without immobilization) under the optimized conditions of NADH before optical glucose biosensor studies using enzyme-immobilized MNPs. First, the enzymatic reaction took place between glucose and GDH in the presence of the NAD^+^ cofactor at pH 7.0, 1.0 M NH_4_CH_3_COO. To enable the colorimetric reaction between the enzymatically produced NADH and [Cu(Nc)_2_]^2+^, the CUPRAC reagent was added to the solution after 20 min for the completion of the enzymatic reaction. Finally, the absorbance of the yellow [Cu(Nc)_2_]^+^ complex formed as a result of the colorimetric reaction was measured at 450 nm. A 20 min enzymatic reaction time, 10 μL of 2.0 mg/mL GDH, 30 °C temperature, 1.0 M NH_4_CH_3_COO with pH 7.0, and a 5 min colorimetric reaction were optimized by monitoring the absorbance of the formed complex based on two glucose concentrations of 50.0 and 100.0 μM. The curves of each optimization parameter are given in the Supporting Information file (optimization I, Figure S8). In addition, the spectra, photographs, and calibration curves of linear and nonlinear regions are given in Figure S9. As seen in this figure, the intensity of the yellow complex and the peaks at 450 nm increased depending on the glucose concentration in the range between 1.0 and 200.0 μM. The linear concentration range was found to be between 1.0 and 150.0 μM [*A* = 0.0124*C* (μM) + 0.0475].

**Figure 3:**
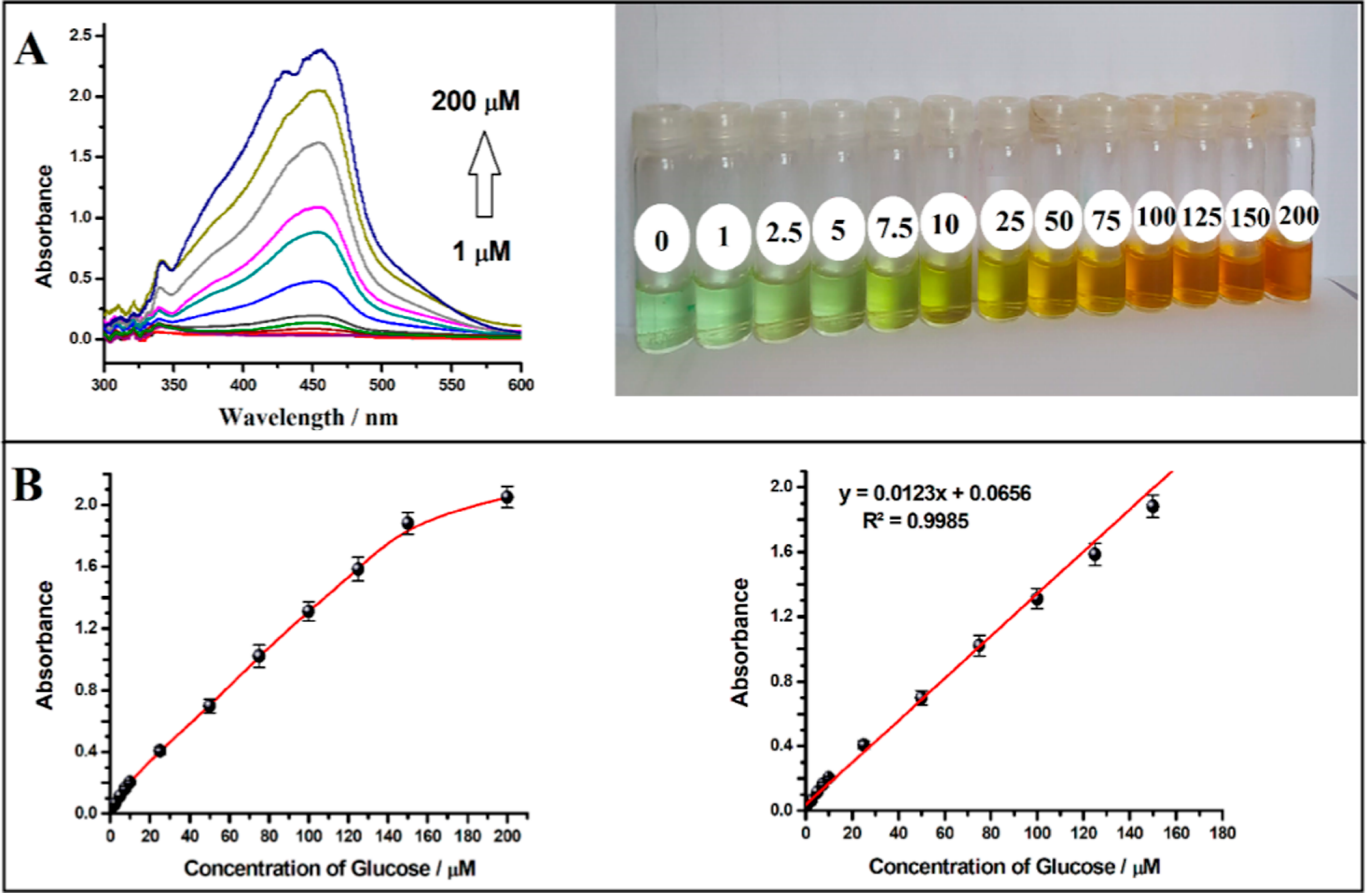
(A) Spectra and photographs of [Cu(Nc)_2_]^+^ formed as a result of the reaction between [Cu(Nc)_2_]^2+^ and NADH, which was liberated by the enzymatic (using GDH@CS@SiO_2_@Fe_3_O_4_ NPs) reaction of different concentrations of glucose in the presence of 10 mM NAD^+^. (B) Curves obtained from nonlinear and linear regions based on absorbance values recorded at 450 nm for glucose at different concentrations.

In the next step, the enzymatic reaction between glucose and GDH was performed using GDH-immobilized MNPs (GDH@CS@SiO_2_@Fe_3_O_4_ NPs). In this context, optimization studies for the amounts of enzyme and SiO_2_@Fe_3_O_4_ NPs during the immobilization of the enzyme and the enzymatic reaction time were performed; the obtained results are given in the Supporting Information file as optimization II (Figure S10). Under optimal conditions (0.40 mg of GDH immobilized on 15 mg of SiO_2_@Fe_3_O_4_ NPs and a reaction time of 30 min), enzymatic reactions were carried out with increasing glucose concentrations from 1.0 to 200.0 μM using GDH@CS@SiO_2_@Fe_3_O_4_ NPs. Then, MNPs were separated with a magnet, and the colorimetric reaction was performed to obtain the spectra and to measure absorbances as described before. The spectra, photographs, and linear and nonlinear calibration curves are given in Figure 3. Similar to enzyme-free studies, the increments in the color intensity of the complex and the peaks at 450 nm, depending on glucose concentration, were also obtained with GDH@CS@SiO_2_@Fe_3_O_4_ NPs. In Figure 3B, the linear equation obtained from the calibration curve of glucose was found to be *A* = 0.0123*C* (μM) + 0.0656. The linear range was found to be 1.0–150.0 μM, and the LOD and LOQ values were 0.31 and 1.02 μM by using the expressions 3*s*_b_/*m* and 10*s*_b_/*m*, respectively. The RSD values of 2.2 and 4.7% for 10 and 100 μM glucose calculated from three different absorbance measurements indicate that the designed glucose biosensor has very good repeatability. The slope obtained for the designed biosensor was found to be very close to the slope obtained for the glucose biosensor based on the use of the enzyme in solution medium (0.0124) and the slope obtained for pure NADH (0.0144). This result supports the hypothesis that in the presence of GDH immobilized on the SiO_2_@Fe_3_O_4_ surface, NADH is formed proportionally to the glucose concentration, and the formed NADH reacts with [Cu(Nc)_2_]^2+^ to form the yellow-colored [Cu(Nc)_2_]^+^ chromophore.

**Figure 4:**
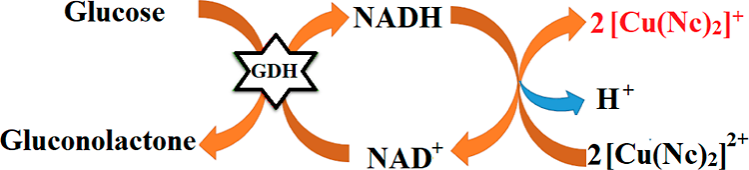
Mechanism of the optical glucose biosensor comprises the GDH enzyme and the CUPRAC reagent.

The mechanism of the optical biosensor depending on GDH is presented in Figure 4. In the first step, an enzymatic reaction takes place between glucose and GDH on SiO_2_@Fe_3_O_4_ in the presence of NAD^+^. At the end of the enzymatic reaction, NAD^+^ is reduced to NADH, while glucose oxidizes to gluconolactone. After GDH@CS@SiO_2_@Fe_3_O_4_ NPs are separated with a magnet, a second reaction takes place between the CUPRAC reagent and enzymatically produced NADH. In this colorimetric reaction, light blue [Cu(Nc)_2_]^2+^ is reduced to yellow [Cu(Nc)_2_]^+^, of which the color intensity is increased proportionally with glucose concentration, while enzymatically produced NADH is oxidized to NAD^+^. Thus, the glucose biosensor operates on the basis of the absorbance measurement of the yellow-orange [Cu(Nc)_2_]^+^ complex at 450 nm.

Table 1 shows a comparison of the analytical figures of merit of the proposed biosensor with those reported in some previously published optical glucose biosensors. Although the sensitivity of some other methods, such as amperometric^5^ (LOD: 0.04 μM) and electrochemiluminescence^47^ (LOD: 1.2 nM), is better than that of the proposed GDH–CUPRAC-based glucose biosensor, the sensitivity of the current method is close to those of colorimetric methods and even better than some of them. As seen in Table 1, colorimetric glucose biosensor studies based on different strategies have been carried out. The most commonly used among them is the colorimetric sensor based on the formation and measurement of the absorbance of TMB with different nanomaterials showing peroxidase-like properties.^48−52^ Compared to these methods, the LOD value of the novel CUPRAC–GDH-based biosensor was generally found to be close to or even lower than the values reported in some of these studies. It is seen that the LOD value of the GDH–CUPRAC-based biosensor was also found to be lower than those of biosensors based on ABTS,^53,54^ I_2_-starch,^55^*o*-dianisidine,^56^ FeSCN^2+^ including Fenton reaction,^57^ and CUPRAC^41^ reagents, which is quite similar to our study but using the GOx enzyme. Moreover, the LOD value of the GOx-based glucose biosensor is lower than or comparable with those of other strategies based on etching^58,59^ or formation^60^ of plasmonic metal nanoparticles. The proposed colorimetric reaction has a definite stoichiometry between Cu(Nc)_2_^2+^ and NADH, whereas the colorization of redox-active dyes, such as TMB, does not have a clear stoichiometry. Moreover, the linear range of the current method covering more than 2 orders of magnitude is quite wide. This novel biosensor, having more sensitivity and clarity of reaction stoichiometry than other similar assays, is expected to find further use in biosensor design.

**Table 1:** Comparison of the Analytical Performance of the Constructed Biosensor with Those of Related Colorimetric Biosensors^a^

method	C/W (nm)	LR (μM)	LOD (μM)	refs
CR between Enzymatically Produced H_2_O_2_ and TMB_red_ Using GOx and Peroxidase-Mimic Catalysts				
(i) core–shell Cu/Au NPs	blue TMB_ox_/652–653	20–670	15	(48)
(ii) C60-carboxyfullerene, C_60_[C(COOH)_2_]_2_	—	1–40	0.5	(49)
(iii) Co_3_O_4_@CeO_2_ hybrid microspheres	—	1–75	1.9	(50)
(iv) Si-dots	—	0.17–200	0.05	(51)
(v) AgNPs@rGO	—	125–1000	40	(52)
CR between Enzymatically Produced H_2_O_2_ and ABTS Using GOx and below Peroxidase-Mimic Catalysts				
(i) PDDA-coated Fe_3_O_4_	green ABTS^+•^/420	30–100 and 200–1000	30	(53)
(ii) CMC-PB NPs	—	5–100	1.0	(54)
CR between Enzymatically Produced H_2_O_2_ Using GOx and below Materials				
(i) KI using gelatin and starch	blue I_2_-starch/570	500–5000	50	(55)
(ii) *o*-D_red_ and HRP	red *o*-D_ox_/–	100–500	30	(56)
(iii) Fenton reaction and FeSCN^+^	red FeSCN^2+^/470	10–4000	10	(57)
CR between Metal Nanoparticles and H_2_O_2_ Using GOx				
(i) AgTNPs (etching)	from blue to purple/600	0–1000	10	(58)
(ii) Au@Ag NPs (etching)	from orange to wine red/375	0.5–400	0.24	(59)
(iii) Au–Ag core–shell NPs (formation)	from red to yellow/410	40–1000	0.01	(60)
CR between CUPRAC and H_2_O_2_ Using GOx				
Fe_3_O_4_/SiO_2_/APTS/GAL/GOx	yellow [Cu(Nc)_2_]^+^/450	11.1–111.1	0.59	(41)
CR between CUPRAC and NADH Using GDH				
GDH@CS@SiO_2_@Fe_3_O_4_ NPs	yellow [Cu(Nc)_2_]^+^/450	1.0–150	0.31	this work

a Abbreviations: C: chromogenic reagent; CR: colorimetric reaction; PDDA: poly(diallyldimethylammonium chloride); GOx: glucose oxidase; TMB: 3,3′,5,5′-tetramethylbenzidine; ABTS: 2,2′-azinobis(3-ethylbenzothiazolinesulfonic acid)diammonium salt; APTS: (3-aminopropyl)triethoxysilane; GAL: glutaraldehyde; AgTNPs: silver triangular nanoplate; W: wavelength; LR: linear range; LOD: limit of detection; PB: Prussian blue; CMC: carboxymethyl cellulose; *o*-D: *o*-dianisidine; red: reduced form and ox: oxidized form.

### 3.4 Interference Studies

The interference effects of other monosaccharides and some possible interfering molecules, such as ascorbic acid (AA), dopamine (DA), and uric acid (UA), on the designed optical glucose biosensor were investigated. For this purpose, enzymatic and colorimetric reactions of each of the analyte-free interfering molecules (50 μM) and solutions containing analyte/interferent at a ratio of 1:1 or 10:1 were performed under optimized conditions, followed by absorbance measurements at 450 nm. Table S1 shows that other monosaccharides and sucrose did not exhibit any interference effect on the glucose response as expected, but DA, AA, and UA gave significant positive interference even at a 10:1 analyte/interferent ratio. In order to eliminate interferences of AA, DA, and UA, the biosensing study was carried out after the solutions were passed through a syringe filled with a preoxidant (∼0.5 g NaBiO_3_), as previously applied in our amperometric glucose biosensor study.^44^ While the preoxidant cannot oxidize glucose, positive interferences of DA, AA, and UA were effectively suppressed since these interfering compounds are easily oxidized by NaBiO_3_ before the enzymatic and colorimetric reactions.

### 3.5 Stability Studies

**Figure 5:**
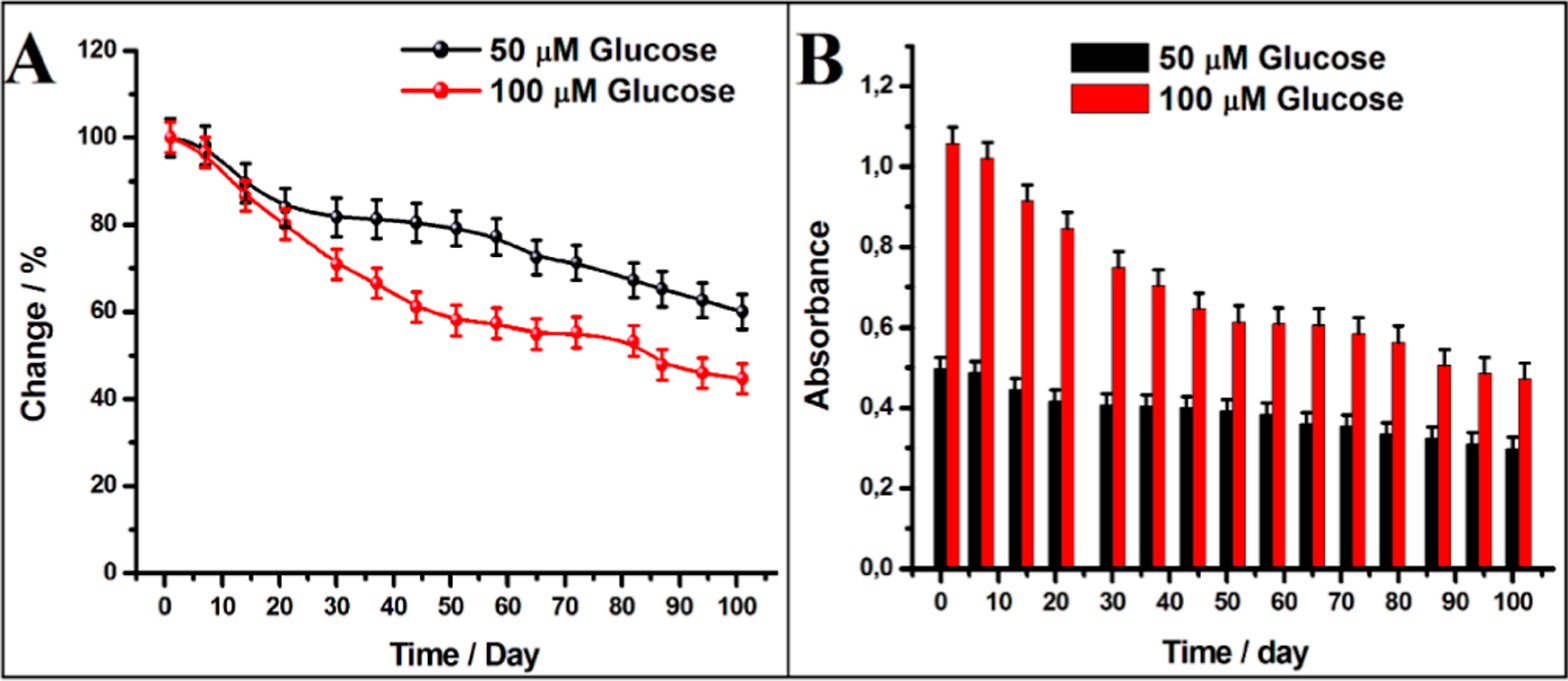
Long-term stability of the optical glucose biosensor. Graphs of absorbance (A) and percentage absorbance change (B) versus time (day), respectively.

To investigate the stability of the developed optical glucose biosensor, the absorbance at 450 nm was recorded after the enzymatic and colorimetric reactions of the glucose solution at two different concentrations (50.0 and 100.0 μM) every 7 days for approximately 100 days. When the biosensor was not used, the GDH-immobilized MNPs were washed with pH 5.0 PBS after each measurement and stored in a refrigerator at 4 °C in a slightly humidified medium. The graph of the change in absorbance recorded every 7 days for 100 days and the graph of the decrease in absorbance compared to the first measurement are shown in Figure 5. While no significant decrease in absorbance was observed in the first week, it was observed that there was a slight decrease in absorbance after the 7th day, and this decrease was more apparent for a 100.0 μM solution (up to 80% at the end of the 20th day and up to 50% at the end of the 100th day). On the other hand, for a 50 μM solution, the absorbance remained almost constant after the 20th day and decreased to 80% of the initial value even on the 100th day. As a result, the designed biosensor was measured once a week, and it was determined that even after 100 days, 80% of the response was maintained for 50 μM and 50% for 100 μM. These data show that the designed biosensor has acceptable stability, the long-term preservation of which can be attributed to the very good stability of the CS film on silica-coated Fe_3_O_4_, its biocompatible environment, and the effective immobilization of GDH, which prevents the leakage of the enzyme.

### 3.6 Real Sample Analysis

To test the applicability of the newly developed optical biosensor, an artificial blood serum sample and various types of real samples, such as beverages, commercial dextrose serum, glucose tolerance test drinks, and human blood serum, were analyzed with this biosensor. In addition, recovery studies were carried out by the standard addition of glucose to real blood samples. Before the enzymatic reaction, all diluted samples were passed several times (at least 10) through a syringe containing 0.5 g of NaBiO_3_, to minimize interference coming from AA, DA, and UA. After the enzymatic reaction (30 min) using GDH@CS@SiO_2_@Fe_3_O_4_ NPs under optimized conditions, the colorimetric reaction (5 min) was performed, and the absorbance values of all samples were recorded at 450 nm. The dilution factor was taken into consideration in all calculations. In addition, all samples were also analyzed with a validated enzymatic method based on the spectrophotometric determination of enzymatically produced NADPH at 340 and 700 nm.^45^ In this method, the first enzymatic reaction occurred between hexokinase and glucose in the presence of ATP. Then, NADPH was produced in the second enzymatic reaction, which occurred between G-6-PDH and enzymatically produced G-6-P from the first reaction in the presence of the cofactor NADP^+^. From the results given in Table 2, it can be seen that good agreement was obtained between the fabricated biosensors and hospital results. Moreover, recovery values close to 100% calculated from real samples indicate that the accuracy of the developed optical biosensor is quite good. All these results prove that the applicability of the fabricated optical glucose biosensor based on GDH and CUPRAC reagents is reliable.

**Table 2:** Real Sample Analysis with a Proposed Optical Biosensor and a Validated Spectrophotometric Method

sample	found glucose concentration (mM)	
	fabricated optical biosensor/recovery %	spectrophotometric method (hospital results)
commercial dextrose serum (calculated value: 252.5 mM)	252.7 ± 1.8	269.0 ± 1.0
beverage 1 (fruit juice)	133.7 ± 0.8	135.3 ± 4.0
beverage 2 (ice tea)	135.7 ± 2.3	156.0 ± 2.3
glucose tolerance test drink (calculated value: 1514)	1547 ± 4	1764 ± 4
artificial blood serum (containing 4.77 mM G)	4.6 ± 0.2	4.9 ± 0.4
human serum 1	5.3 ± 0.1	5.2 ± 0.1
human serum 1 + 2.5 mM G^a^	8.0 ± 0.2/102.4	
human serum 1 + 5.0 mM G^a^	11.1 ± 0.2/107.6	
human serum 2	5.2 ± 0.1	5.1 ± 0.2
human serum 2 + 2.5 mM G^a^	7.3 ± 0.2/94.8	
human serum 2 + 5.0 mM G^a^	11.2 ± 0.3/110.4	
human serum 3	5.6 ± 0.2	5.4 ± 0.2
human serum 3 + 2.5 mM G^a^	7.7 ± 0.1/95.1	
human serum 3 + 5.0 mM G^a^	11.5 ± 0.1/108.2	

a Spiked human blood samples with a given concentration of glucose (G).

## 4 Conclusions

The CUPRAC reagent is a useful chromogenic oxidant that has led to the development of many optical sensors, especially for the determination of TAC. In this study, the colorimetric reaction of the CUPRAC reagent with NADH, emerging as the reaction product of dehydrogenase enzymes, was investigated for the first time. As a result of this study, an optical glucose biosensor based on the use of the CUPRAC reagent and GDH-immobilized silica-coated MNPs was successfully designed. Although a biosensor based on the glucose oxidase enzyme has been developed in the literature, a biosensor study based on the CUPRAC reagent with the dehydrogenase enzyme has not yet been performed. The incorporation of the CUPRAC reagent into the designed biosensor assures a clear stoichiometry and a wide linear range arising from single product (cuprous neocuproine) colorimetry, minimizing chemical deviations from Beer’s law. Therefore, the first-time integration of the CUPRAC reagent into the dehydrogenase enzyme-based biosensor reflects the novelty of this study. The LOD value of the developed optical biosensor using GDH-immobilized SiO_2_@Fe_3_O_4_ was found to be 0.31 μM, which reflects its high sensitivity. The interference effects of some molecules such as AA, DA, and UA, which also give the colorimetric reaction with the CUPRAC reagent along with the enzymatic reaction product of NADH, were eliminated with a preoxidant (NaBiO_3_) before the enzymatic reaction. The precision of the developed biosensor was quite good, and its stability was found to be acceptable after about 3 months. The designed optical glucose biosensor has been successfully applied to beverages, glucose test solutions, dextrose serum solutions, and real blood samples. Moreover, it has acceptable accuracy because recovery values close to 100% were obtained from glucose-spiked human serum blood samples. It is predicted that the CUPRAC reagent can be used for different analytes using various dehydrogenase enzymes or that it may pave the way to the development of optical biosensors for various analytes by utilizing the reaction of this reagent with enzymatic reaction products based on other enzymes.

## Supporting Information Available

Reagents and apparatus, synthesis of silanized Fe_3_O_4_ NPs, and immobilization of GDH on SiO_2_@Fe_3_O_4_ NPs (PDF)
