# Supplementary material for: Fabrication of a Novel Optical Glucose Biosensor Using Copper(II) Neocuproine as a Chromogenic Oxidant and Glucose Dehydrogenase-Immobilized Magnetite Nanoparticles
Source: ACS Omega. 2023 Dec 2;8(49):47163–72. doi: 10.1021/acsomega.3c07181 (PMC10719923; doi:10.1021/acsomega.3c07181)
Supplement: Supplementary file 1 — ao3c07181_si_001.pdf [file ao3c07181_si_001.pdf]

Supporting Information

Fabrication of a Novel Optical Glucose Biosensor Using Copper (Ii)-Neocuproine as a Chromogenic Oxidant and Glucose Dehydrogenase-Immobilized Magnetite Nanoparticles

Selen Ayaz[1], Ayşem Üzer[2], Yusuf Dilgin*[1], Reşat Apak*[2]

[1]*Canakkale Onsekiz Mart University, Faculty of Science, Department of Chemistry, Canakkale, Turkey*

[2]*İstanbul University -Cerrahpaşa, Faculty of Engineering, Department of Chemistry, İstanbul-Avcılar, Turkey*

Contents	Page
**1.** Experimental section	S2
**1.1.** Reagents and apparatus	S2
**1.2.** Synthesis of Silanized Fe_3O_4 NPs	S2
**1.3.** Immobilization of GDH on $SiO_2@Fe_3O_4$ NPs	S2
**Scheme S1:** Procedure for Immobilization of GDH on $SiO_2@Fe_3O_4$ NPs	S3
**2.** Characterization of Synthesized and Enzyme Immobilized Fe_3O_4 NPs	S3
**Fig. S1.** TEM images	S4
**Fig. S2.** FTIR spectra	S5
**Fig. S3.** SEM images	S6
**Fig. S4.** EDX spectra	S7
**Fig. S5.** Elemental mapping	S7
**Fig. S6.** Elemental mapping	S8
**Fig. S7**. XRD patterns	S8
**3.** Optimization Study I (Free enzyme in the solution).	S8
**Fig. S8.** Optimization curves	S9
**4.** Studies on Optical Glucose Biosensor using GDH in solution (Free enzyme)	S10
**Fig. S9. A)** Spectra and photographs of $[Cu(Nc)_2]^+$ and **B)** Calibration curves	S10
**5.** Optimization study II:	S10
**Fig. S10.** Optimization curves	S11
**6.** Interference study	S12
**Table S1.** Results obtained from interference study for glucose biosensor based on the use of GDH immobilized $SiO_2@Fe_3O_4$ NP	S12
**7.** References	S13

1. Experimental Section

1.1. Reagents and apparatus

Main chemicals such as β-Nicotinamide adenine dinucleotide sodium salt (NAD^+), glucose dehydrogenase from *Pseudomonas sp.* (GDH, powder, white, ≥200 U/mg), neocuproine (2,9-Dimethyl-1,10-phenanthroline), $CuCl_2$, glutaraldehyde (GAL, 25% in H_2O), chitosan, (3-aminopropyl) triethoxysilane (APTES), tetraethyl orthosilicate (TEOS), $FeCl_3.6H_2O$, $FeSO_4.7H_2O$, NH_4OH, and NH_4CH_3COO were purchased from Sigma Aldrich, or Merck, or Fluka companies. All other reagents were used in their analytical grade forms.

A Hanna 221 pH-meter containing a Hanna 220 probe was used to measure the pH of the solutions. An Ocean Insight brand HR4Pro spectrophotometer containing a light source of Ocean Insight DH-2000-BAL UV/VIS-NIR and Shimadzu brand UV-1208 UV-VIS were used for recording spectra and absorbance measurements.

1.2. Synthesis of Silanized Fe_3O_4 NPs

Fe_3O_4 NPs were prepared using a procedure reported in the literature[1] by dissolving 3.06 g of $FeSO_4.7H_2O$ and 5.94 g of $FeCl_3.6H_2O$ in 100 mL of 2.0 M HCl and then adding NH_4OH until the pH of the solution reached 9.0 after passing the Ar through the solution for 10 min to remove O_2. Afterward, the prepared solution was shaken at 80 ^oC for 60 min under an Ar atmosphere. The black-colored Fe_3O_4 that formed was separated by a magnet and washed with pure water until it reached a neutral pH. Then, it was washed with ethanol and dried in a vacuum oven at 50 ^oC for silanization.

For silanization, 1.0 g of Fe_3O_4 was coated with TEOS in the presence of CTAB using the procedure reported by Ahangaran et al.[2] Then, 1.0 g of Fe_3O_4/TEOS nanoparticles were coated with APTES using the procedure reported by Cui et al.[3] The resulting products were then magnetically separated by a magnet and thoroughly washed with ethanol and deionized water until they reached a neutral pH. Finally, APTES-TEOS-Fe_3O_4 nanoparticles (SiO_2@Fe_3O_4 NPs) were kept in a vacuum at room temperature.

1.3. Immobilization of GDH on SiO_2@Fe_3O_4 NPs

Although the surface of magnetite nanoparticles was modified with APTES containing an amine group, chitosan (CS) was also modified onto SiO_2@Fe_3O_4 NPs for more effective enzyme immobilization.[4] Thus, enzyme immobilization was performed by making some changes from the methods used in the literature.[4,5] In the first step, 15 mg SiO_2@Fe_3O_4 NPs were added to 5.0 mL of 1.0% CS prepared in diluted CH_3COOH (pH 4.0), stirred at 150 rpm for 1 hour, separated with a magnet, and thoroughly washed with distilled water. In the second step, 1.0 mL of pH 5.0 PBS and 300 µL of glutaraldehyde (GAL) were added to the magnet-separated nanoparticles, followed by 1 hour of stirring at 150 rpm, magnetic separation, and washing with pH 5.0 PBS, respectively. In the third step, 1.0 mL of pH 5.0 PBS and an optimized amount of enzyme (0.4 mg for GDH) were added to the GAL-treated nanoparticles, followed by 1 hour of stirring at 150 rpm, magnetic separation, and washing with pH 5.0 PBS, respectively. In the last step, enzyme-immobilized nanoparticles (GDH@CS@SiO_2@Fe_3O_4 NPs) were made ready for use by washing with 1.0% BSA (with stirring at 150 rpm for 1 hour), magnet separation, and washing with pH 5.0 PBS to remove nonspecific enzymes adsorbed on the surface. The procedure used is summarized in Scheme S1.

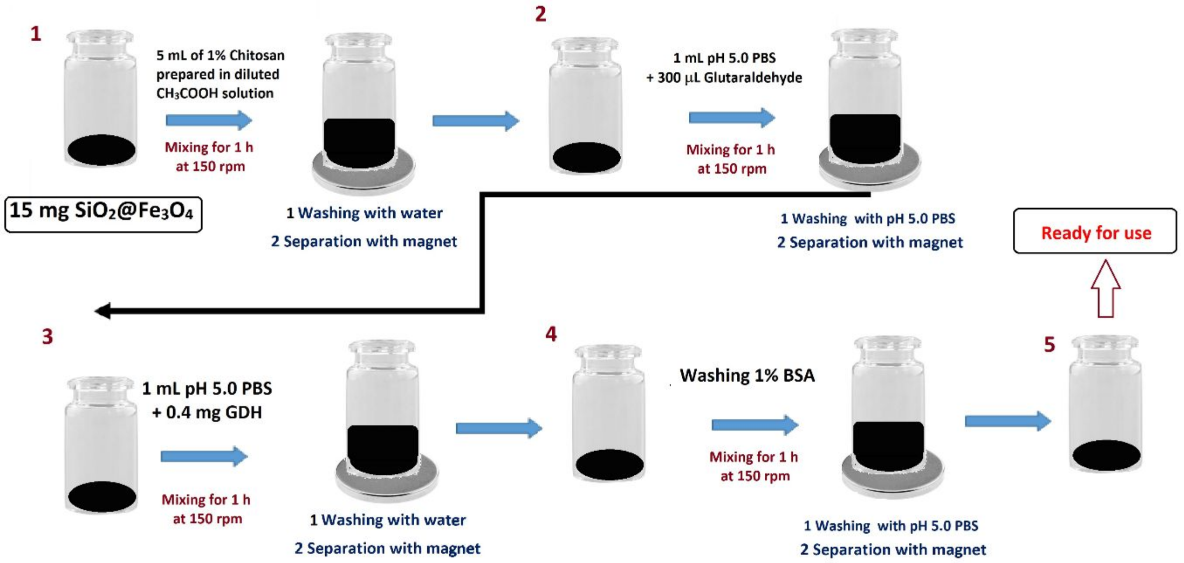

Scheme S1: Procedure for Immobilization of GDH on $SiO_2@Fe_3O_4$ NPs

To characterize silanized and enzyme-immobilized Fe_3O_4 NPs, TEM and SEM images and spectra of EDX, FTIR, XRD, and XPS were recorded at the ÇOBİLTUM of Çanakkale Onsekiz Mart University (for TEM, and XRD), Manisa Celal Bayar University (for SEM and EDX), and Ege University Application and Research Center for Testing and Analysis (for XPS).

2. Characterization of Synthesized and Enzyme Immobilized Fe_3O_4 NPs

TEM images of bare Fe_3O_4, CTAB-Fe_3O_4, TEOS-CTAB-Fe_3O_4, and APTES-TEOS-CTAB-Fe_3O_4($SiO_2@Fe_3O_4$) are shown in **Fig. S1.** The spherical and uniform magnetite nanoparticles were obtained for bare Fe_3O_4 and CTAB-Fe_3O_4 (Figs. S1 a and b). It was observed that Fe_3O_4 spheres agglomerated and SiO_2 grew after modification of Fe_3O_4 NPs with both TEOS and TEOS-APTES (Figs. S1 c and d). Similar results were obtained from previous reported studies.[2,6]

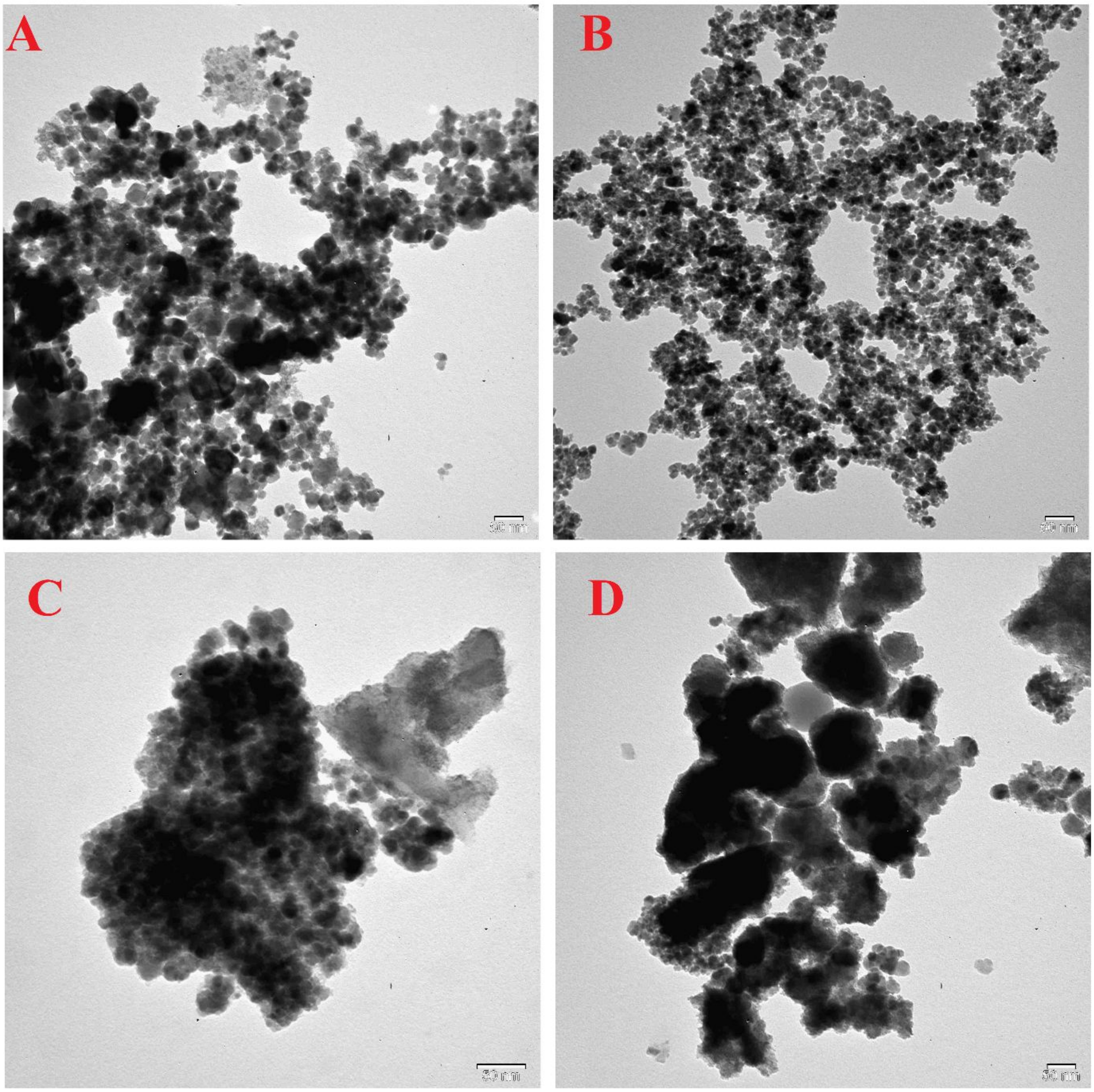

Fig. S1. TEM images of **A)** bare Fe_3O_4, **B)** CTAB-Fe_3O_4, **C)** TEOS-CTAB-Fe_3O_4, and **D)** APTES-TEOS-CTAB-Fe_3O_4 (SiO_2@Fe_3O_4) NPs

FTIR spectra are shown in **Fig. S2.** As expected, **i)** O-H stretching and bending vibrations at 1634 cm^{-1} and at 3400 cm^{-1} due to adsorbed water on bare Fe_3O_4 NPs (**Fig. S2/a**)[2]; **ii)** two aliphatic C-H stretching peaks at 2850 and 2920 cm^{-1} for all CTAB (sharp) **(Fig. S2/b, e, f)**[7], only APTES (weak) (**Fig. S2-c)**[8], and only TEOS (very weak) **(Fig. S2/d)**[6]-; **iii)** C-N stretching and N-H bending peaks at 1520 cm^{-1} and 1487 cm^{-1} for all CTAB and APTES (**Fig. S2/c, e-h)**[7,8]-; and **iv)** sharp peaks attributed to characteristic symmetric and asymmetric Si-O-Si stretching vibrations at 1040 cm^{-1} and 800 cm^{-1} and Si-OH stretching vibrations at 955 cm^{-1} for all APTES- and TEOS-modified Fe_3O_4 NPs (**Fig. S2/c-h)**[2,6] were observed. When GDH was immobilized onto SiO_2@Fe_3O_4 via crosslinking with GAL and CS, two characteristic bands of carbonyl stretching at 1680 cm^{-1} (amid I) and N-H bending at 1520 cm^{-1} (amid II) appeared sharper than each other **(Fig. S2/h)**.[9] All these results prove that GDH is effectively immobilized on magnetite nanoparticle surfaces.

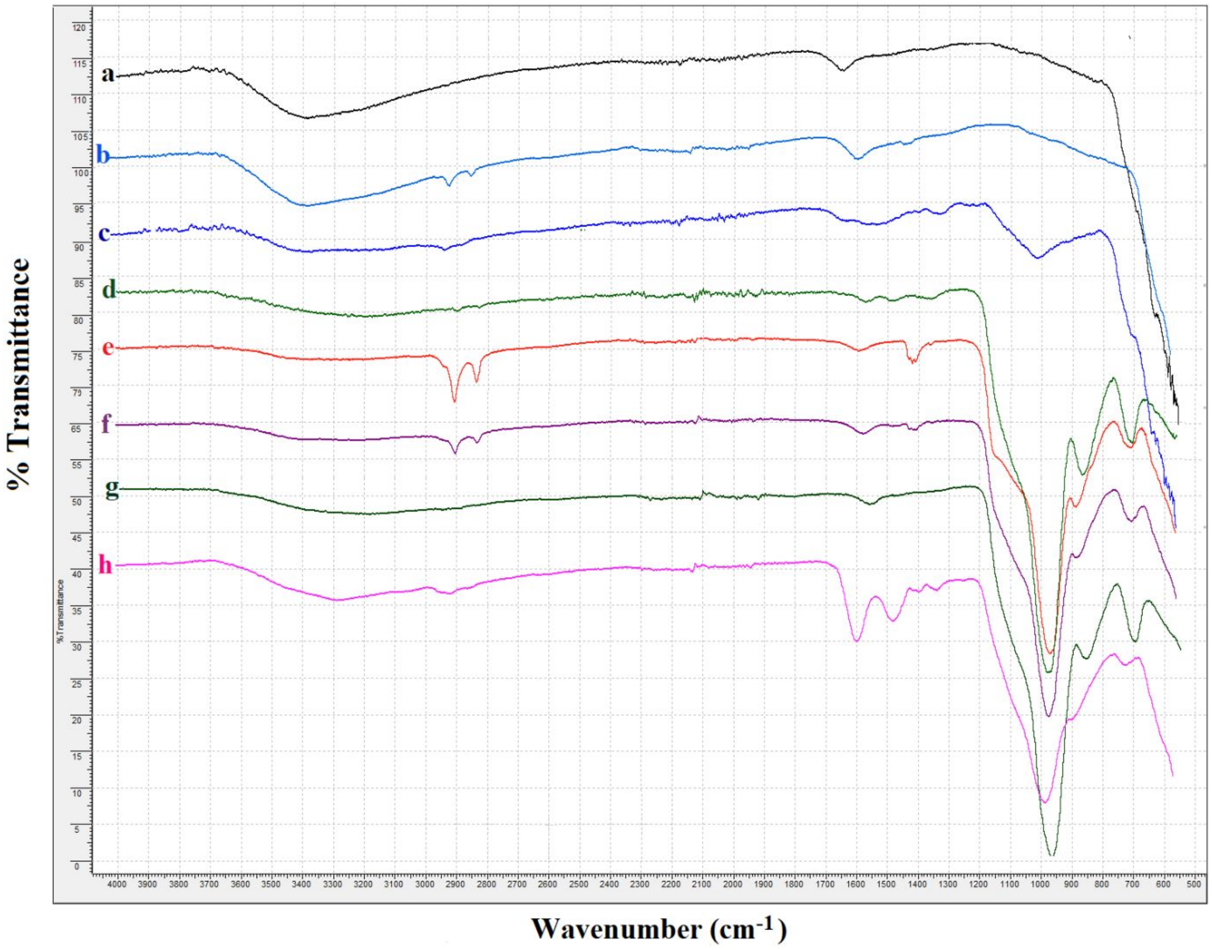

Fig. S2. FTIR spectra of **a)** bare Fe_3O_4, **b)** CTAB-Fe_3O_4, **c)** APTES-Fe_3O_4, **d)** TEOS-Fe_3O_4, **e)** TEOS-CTAB-Fe_3O_4, **f)** APTES-TEOS-CTAB-Fe_3O_4 (SiO_2@Fe_3O_4), **g)** CS@SiO_2@Fe_3O_4, and **h)** GDH@CS@SiO_2@Fe_3O_4 NPs

SEM images of bare Fe_3O_4, TEOS-CTAB-Fe_3O_4, SiO_2@Fe_3O_4, and GDH@CS@SiO_2@Fe_3O_4 were given in **Fig. S3.** While free Fe_3O_4 NPs are in the form of rough sand particles, it is observed that the particles are more homogeneous and turn into a rough spherical structure when coated with CTAB-TEOS and then with APTES. When GDH was immobilized, the morphology of the surface further changed, and an increase in roughness was observed along with agglomeration on the surface. Moreover, EDX spectra (**Fig. S4**) and elemental mapping (**Figs. S5 and S6**) of the magnetite nanoparticles studied were also recorded. As seen in the change in Si atoms' atomic percent in the EDX spectrum (**Fig. S4**) and color distribution in elemental mapping (**Fig. S5**), results were obtained as expected when magnetite nanoparticles were coated with TEOS and followed by APTES. A very small amount of N% in TEOS may be caused by impurities, but the small N% increase with both APTES and then enzyme immobilization is reasonable because of the 1 mole amino group in APTES and the amino acids in the enzyme structure. To support this, the elemental mapping was done by considering only Si and N atoms, and as seen in **Fig. S6**, while N was 9% in SiO_2@Fe_3O_4, it increased up to 11% by enzyme immobilization.

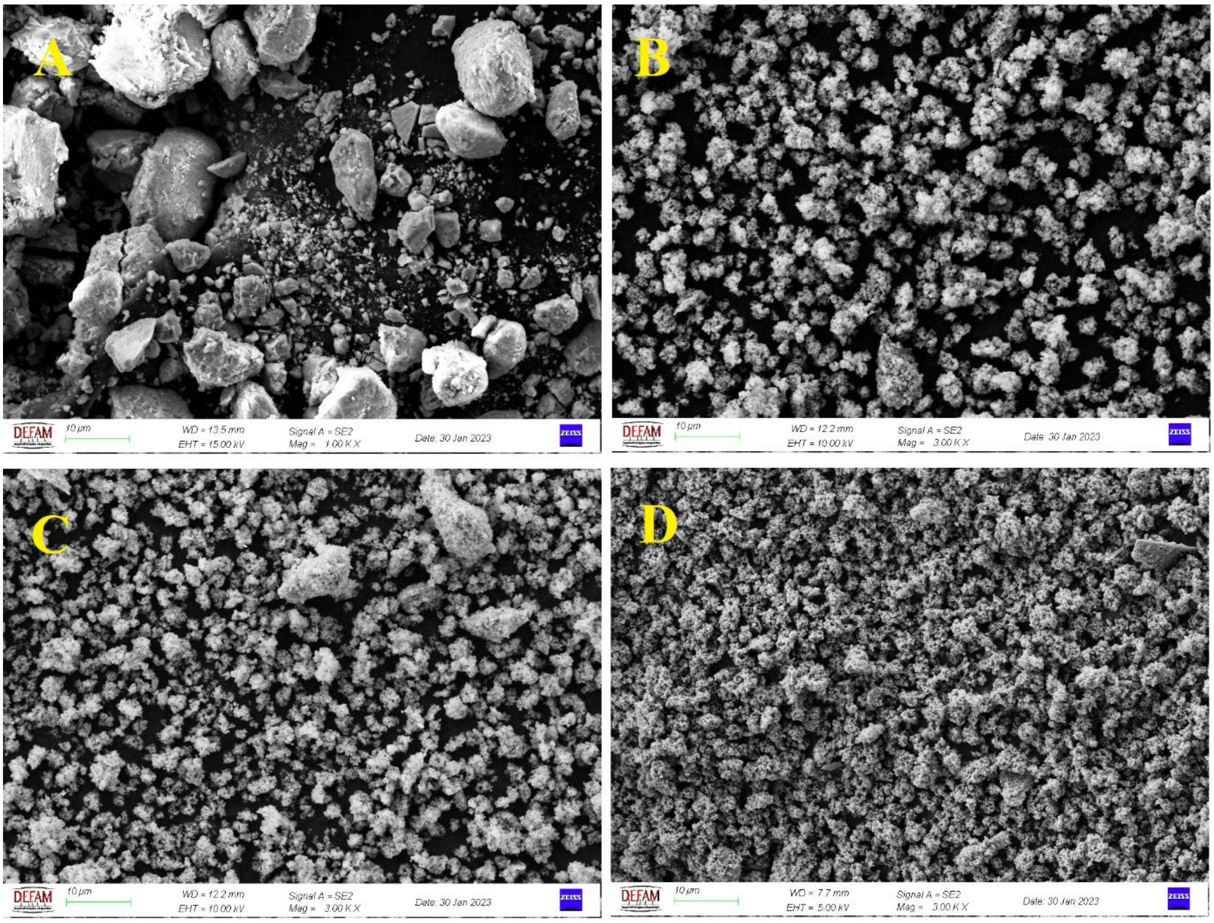

Fig. S3. SEM images of **A)** bare Fe_3O_4 **B)** TEOS-Fe_3O_4 **C)** APTES-TEOS-Fe_3O_4 (SiO_2@Fe_3O_4), and **D)** GDH@CS@SiO_2@Fe_3O_4 NPs

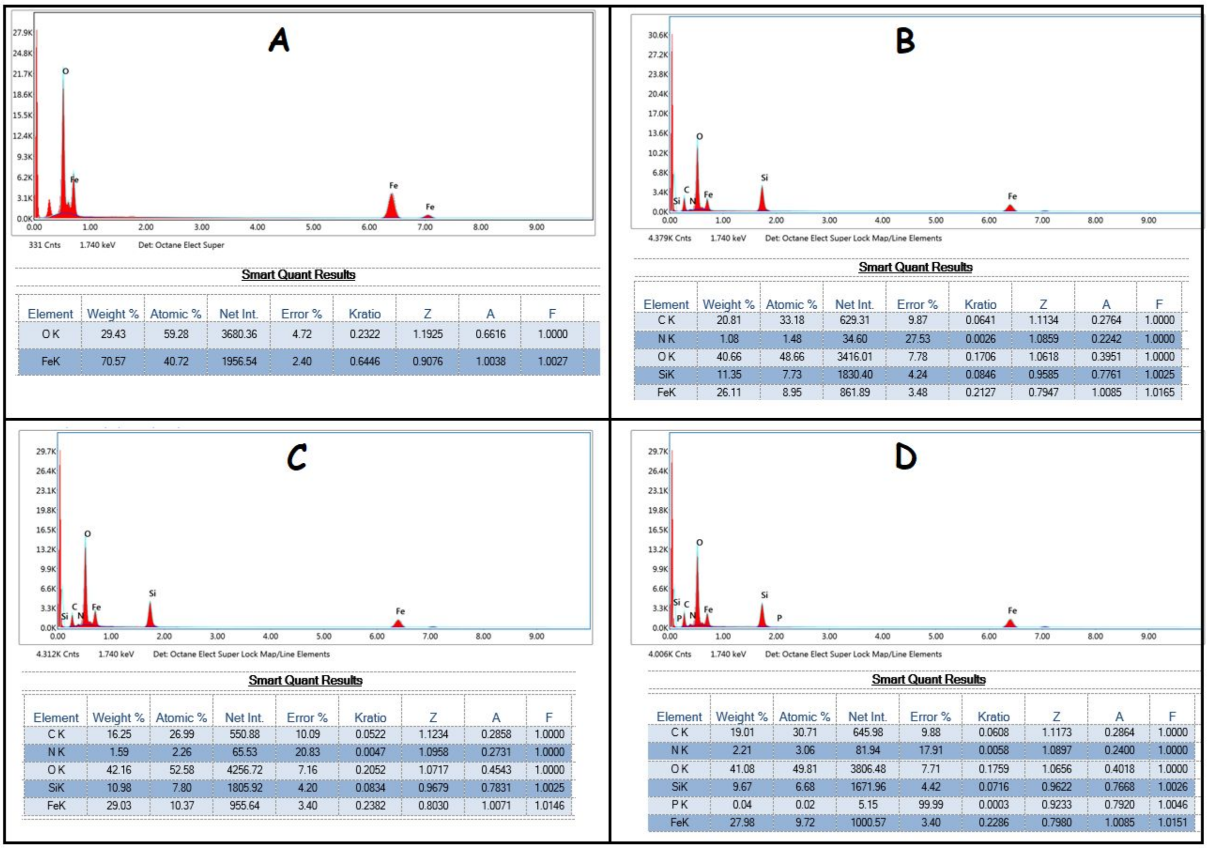

A

Element	Weight %	Atomic %	Net Int.	Error %	Kratio	Z	A	F
O K	29.43	59.28	3680.36	4.72	0.2322	1.1925	0.6616	1.0000
FeK	70.57	40.72	1956.54	2.40	0.6446	0.9076	1.0038	1.0027

B

Element	Weight %	Atomic %	Net Int.	Error %	Kratio	Z	A	F
C K	20.81	33.18	629.31	9.87	0.0641	1.1134	0.2764	1.0000
N K	1.08	1.48	34.60	27.53	0.0026	1.0859	0.2242	1.0000
O K	40.66	48.66	3416.01	7.78	0.1706	1.0618	0.3951	1.0000
SiK	11.35	7.73	1830.40	4.24	0.0846	0.9585	0.7761	1.0025
FeK	26.11	8.95	861.89	3.48	0.2127	0.7947	1.0085	1.0165

C

Element	Weight %	Atomic %	Net Int.	Error %	Kratio	Z	A	F
C K	16.25	26.99	550.88	10.09	0.0522	1.1234	0.2858	1.0000
N K	1.59	2.26	65.53	20.83	0.0047	1.0958	0.2731	1.0000
O K	42.16	52.58	4256.72	7.16	0.2052	1.0717	0.4543	1.0000
SiK	10.98	7.80	1805.92	4.20	0.0834	0.9679	0.7831	1.0025
FeK	29.03	10.37	955.64	3.40	0.2382	0.8030	1.0071	1.0146

D

Element	Weight %	Atomic %	Net Int.	Error %	Kratio	Z	A	F
C K	19.01	30.71	645.98	9.88	0.0608	1.1173	0.2864	1.0000
N K	2.21	3.06	81.94	17.91	0.0058	1.0897	0.2400	1.0000
O K	41.08	49.81	3806.48	7.71	0.1759	1.0656	0.4018	1.0000
SiK	9.67	6.68	1671.96	4.42	0.0716	0.9622	0.7668	1.0026
P K	0.04	0.02	5.15	99.99	0.0003	0.9233	0.7920	1.0046
FeK	27.98	9.72	1000.57	3.40	0.2286	0.7980	1.0085	1.0151

Fig. S4. EDX spectra of **A)** bare Fe_3O_4, **B)** TEOS-CTAB-Fe_3O_4, **C)** APTES-TEOS-CTAB-Fe_3O_4 (SiO_2@Fe_3O_4), and **D)** GDH@CS@SiO_2@Fe_3O_4 NPs

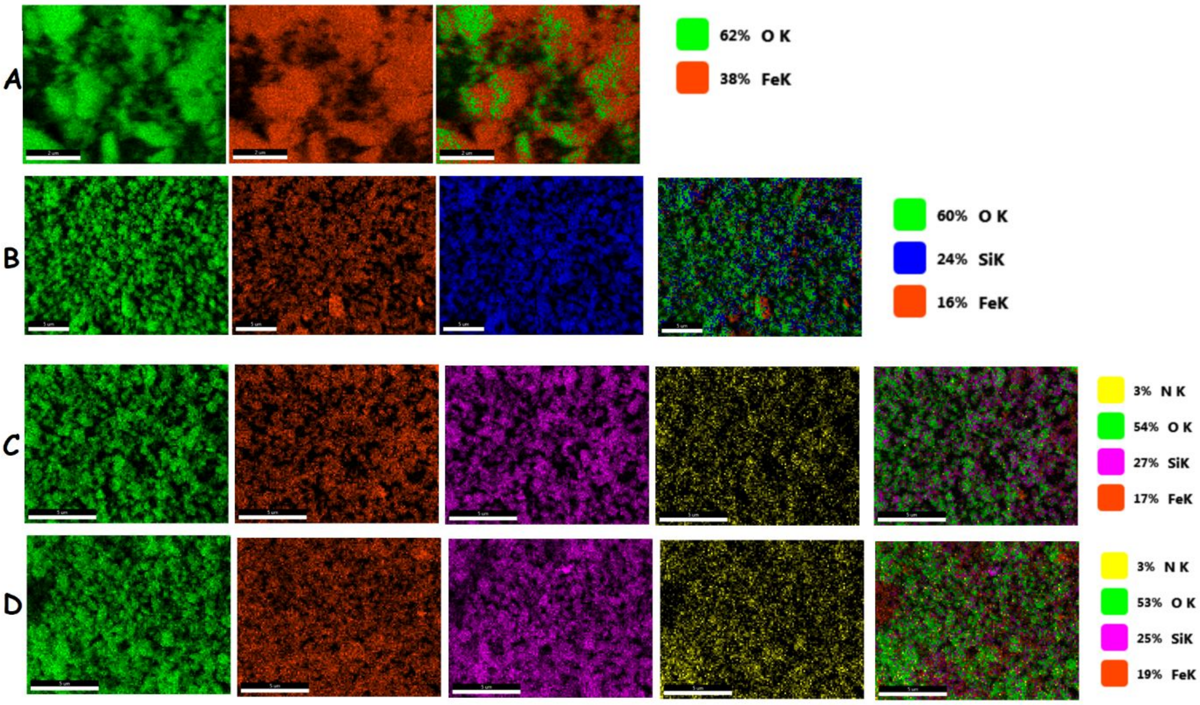

Fig. S5. Elemental mapping of **A)** bare Fe_3O_4, **B)** TEOS-CTAB-Fe_3O_4, **C)** APTES-TEOS-CTAB-Fe_3O_4 (SiO_2@Fe_3O_4), and **D)** GDH@CS@SiO_2@Fe_3O_4 NPs

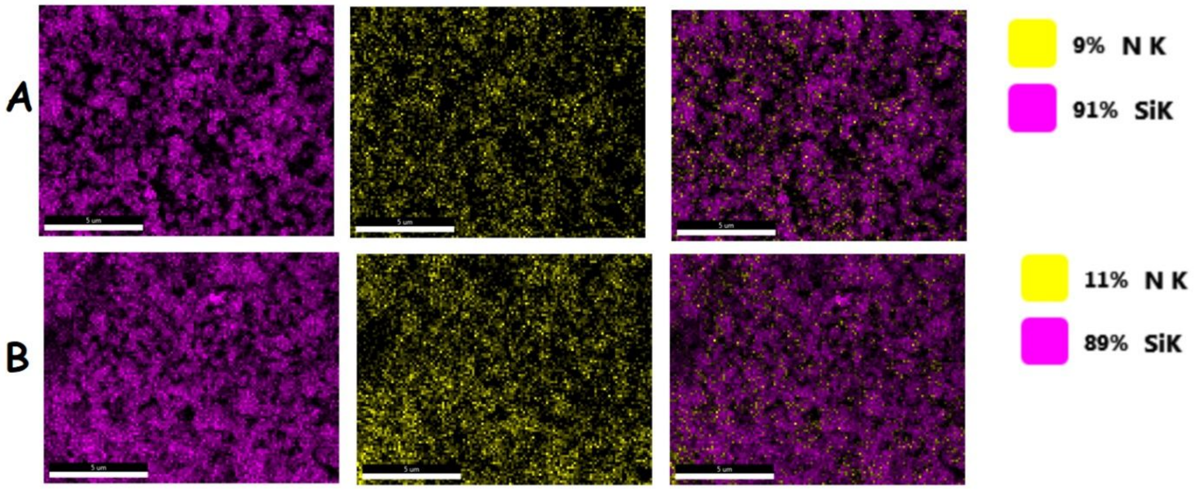

Fig. S6. Elemental mapping of **A)** SiO_2@Fe_3O_4, and **B)** GDH@CS@SiO_2@Fe_3O_4 NPs recorded considering Si and N atoms.

XRD spectra of Fe_3O_4 NPs, Fe_3O_4/TEOS, and Fe_3O_4/TEOS/APTES were also recorded (**Fig. S7**). According to Fig. S7, three samples showed peaks at 2ϴ of 30.43, 35.93, 43.46, 53.62, 57.30, and 62.72°, which can be attributed to the (220), (311), (400), (422), (511), and (440) planes, respectively, of a spinel structure related to Fe_3O_4.[10] Moreover, the XRD patterns before and after modifications of Fe_3O_4 with silanization materials (TEOS and APTES) are similar, indicating that the presence of TEOS and APTES in the nanoparticles does not affect the structure of the support.[11]

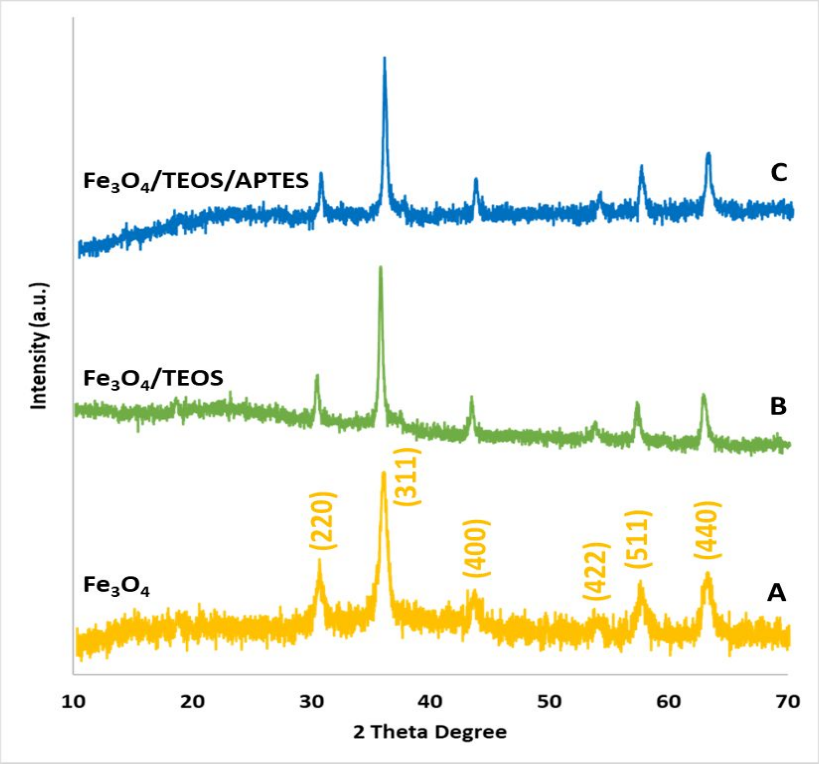

Fig. S7. XRD patterns of Fe_3O_4 (A), Fe_3O_4@TEOS (B) and Fe_3O_4@TEOS@APTES (C)

3. Optimization Study I (Free enzyme in the solution). The enzymatic reaction of glucose with two different concentrations (50.0 and 100.0 μM) was performed using GDH in the presence of 10.0 mM NAD^+. After the enzymatic reaction was completed (about 30 min), the colorimetric reaction was performed on this solution. The absorbance of the $[Cu(Nc)_2]^+$ complex produced from the colorimetric reaction between enzymatically produced NADH and CUPRAC

reagent ($[Cu(Nc)_2]^{2+}$) was monitored depending on the optimized parameter. The curves obtained from optimization studies are given in **Fig. S8,** which shows that maximum absorbance was obtained by using 10 µL of 2.0 mg/mL GDH concentration, a 20-min enzymatic reaction time, a 30 °C temperature, 1.0 M NH_4CH_3COO with pH 7.0, and a 5-min colorimetric reaction time.

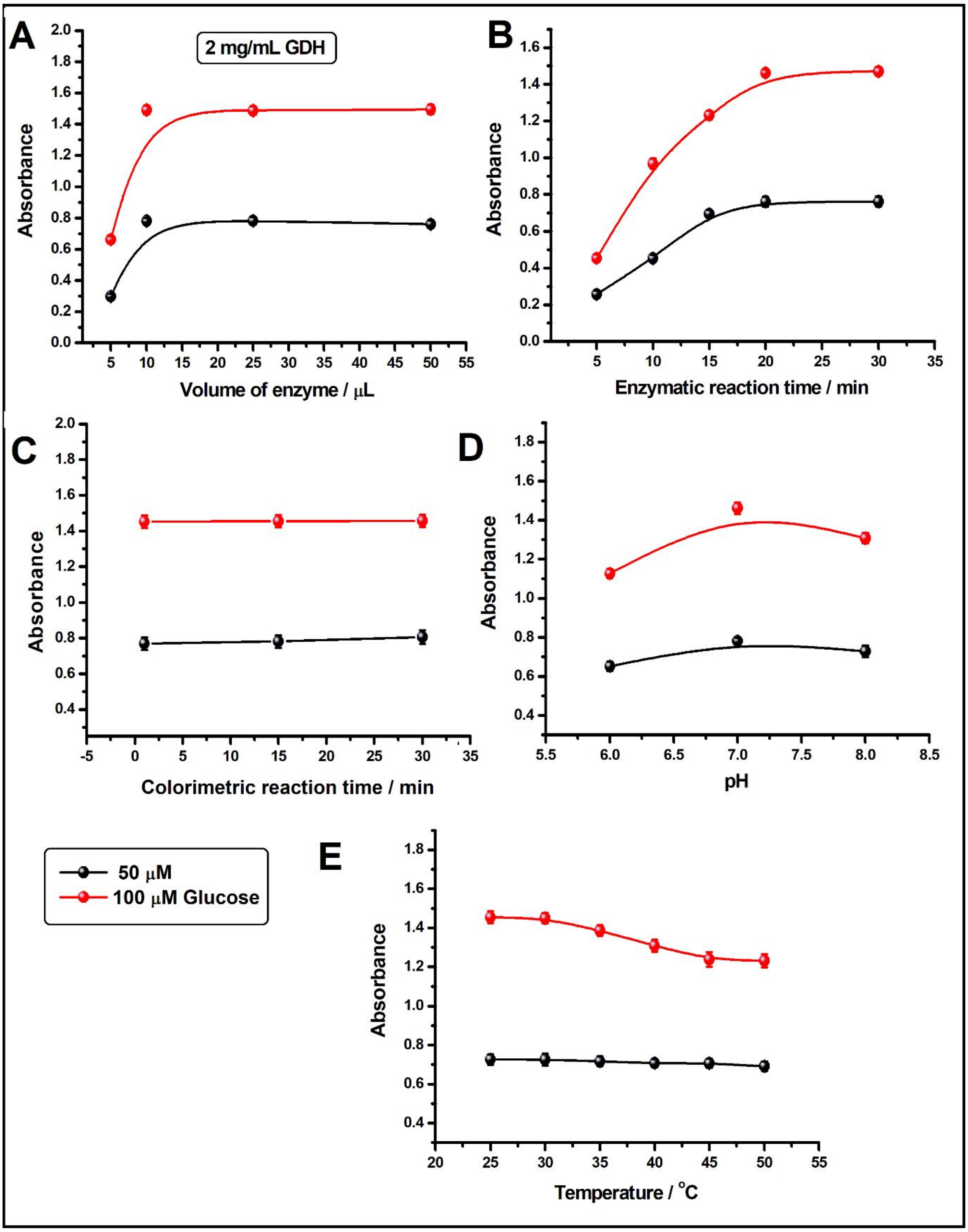

Fig. S8. Optimization curves of **A)** enzyme amount as the volume of a known concentration (2 mg/mL GDH), **B)** enzymatic reaction time, **C)** pH, **D)** temperature, and **E)** colorimetric reaction time, using two different concentrations of glucose (50 and 100 µM)

4. Studies on Optical Glucose Biosensor using GDH in solution (Free enzyme)

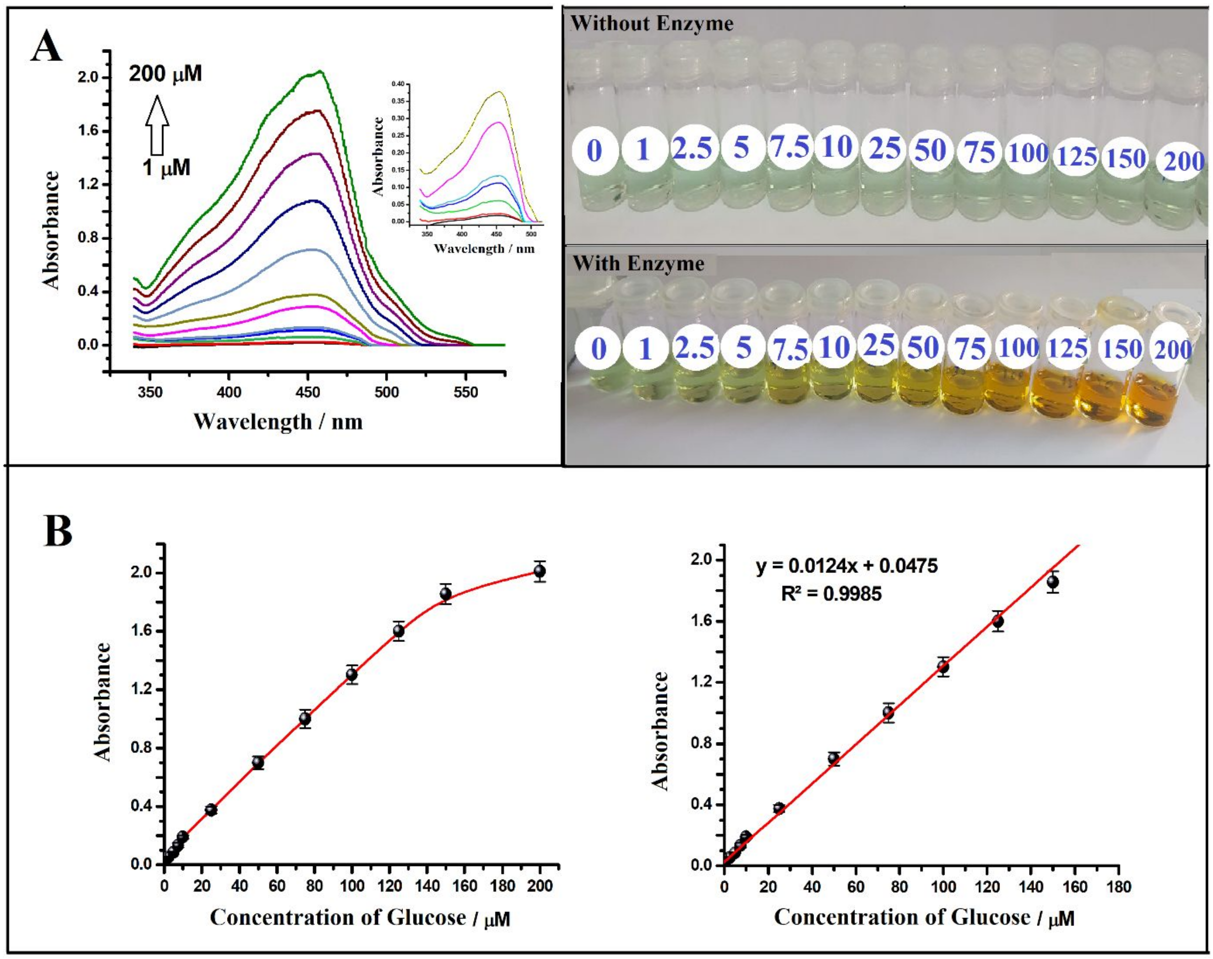

Fig. S9. A) Spectra and photographs of $[Cu(Nc)_2]^+$ formed as a result of the reaction between $[Cu(Nc)_2]^{2+}$ and NADH, which was liberated by the enzymatic (GDH in solution) reaction of different concentrations of glucose in the presence of 10.0 mM NAD^+. **B)** Curves obtained from non-linear and linear regions based on absorbance values recorded at 450 nm for glucose at different concentrations.

5. Optimization study II: The enzymatic reaction of glucose with two different concentrations (50.0 and 100.0 μM) was performed using GDH@CS@SiO_2@Fe_3O_4 NPs in the presence of 10.0 mM NAD^+. After the enzymatic reaction was completed (about 30 min), magnetite nanoparticles were separated with a magnet, and the colorimetric reaction was performed on the solution. The absorbance of the $[Cu(Nc)_2]^+$ complex produced from the colorimetric reaction between enzymatically produced NADH and CUPRAC reagent ($[Cu(Nc)_2]^{2+}$) was monitored depending on the optimized parameter. The curves obtained from optimization studies (**Fig. S10**) show that maximum absorbance was obtained with the use of 0.40 mg enzyme and 15 mg SiO_2@Fe_3O_4 NPs for enzyme immobilization and at least 30 min for the completion of the enzymatic reaction.

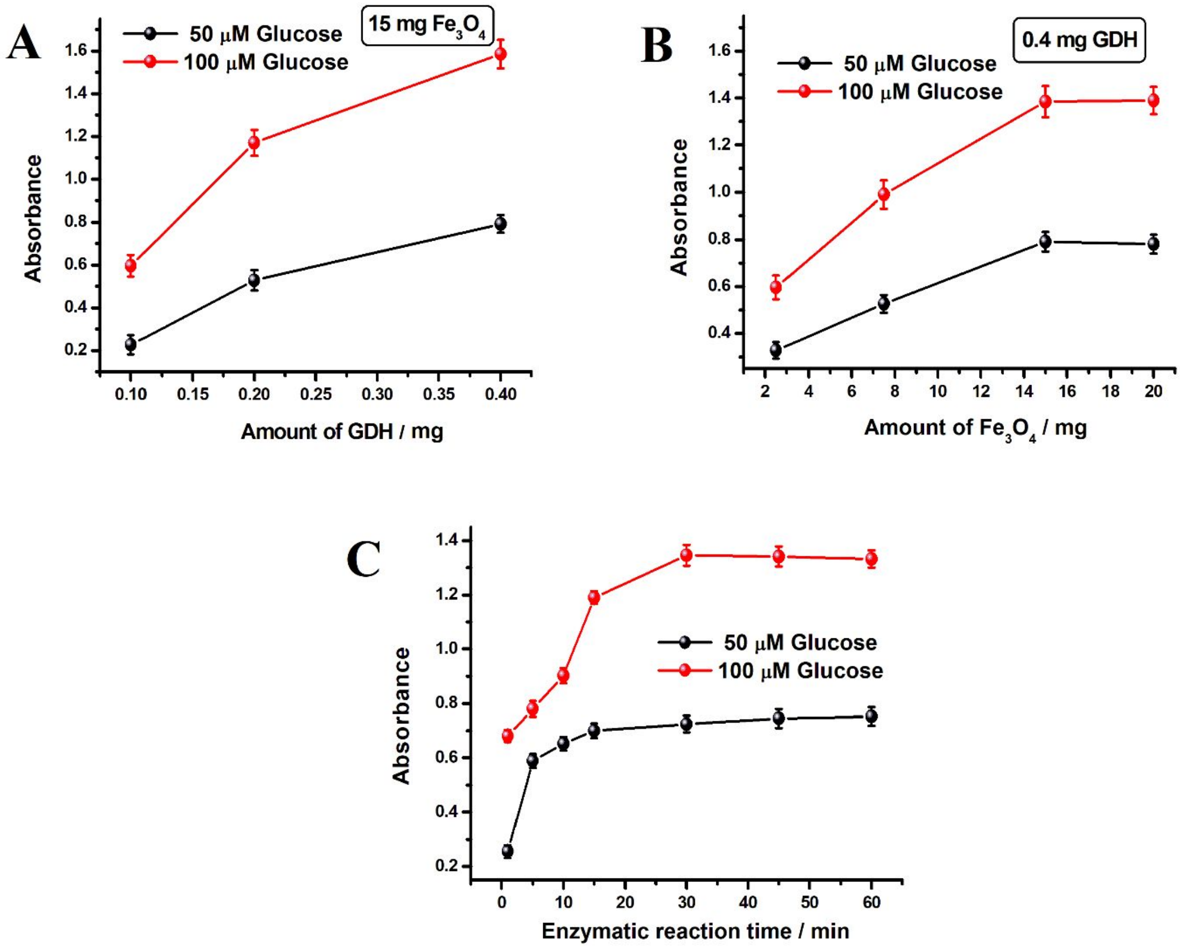

Fig. S10. Optimization curves of **A)** amount of GDH, and **B)** $SiO_2@Fe_3O_4$ NPs in the enzyme immobilization process, and **C)** reaction time during the enzymatic reaction.

6. Interference study

Table S1. Results obtained from the interference study for glucose biosensor based on the use of GDH−immobilized $SiO_2@Fe_3O_4$ NP

**Molecules**	**A_{455} after only CR of 50 µM of species**		**Analyte:Interference**	**ER (30 min) + CR (5 min)**		**Interference %**	
	**Without $NaBiO_3$**	**With $NaBiO_3$**	**G =50 µM Interferences: 50 (for 1:1) or 5 µM (for 10:1)**	**Without $NaBiO_3$**	**With $NaBiO_3$**	**Without $NaBiO_3$**	**With $NaBiO_3$**
Glucose (G)(analyte)	0.021±0.009	-	G	0.759±0.020	0.742±0.015	-	-
Fructose (F)	0.005±0.001	-	1:1 G:F	0.746±0.015	-	-	-
Maltose (M)	0.017±0.006	-	1:1 G:M	0.753±0.018			
Galactose (GA)	0.008±0.002	-	1:1 G:GA	0.743±0.014			
Lactose (L)	0.009±0.003	-	1:1 G:L	0.761±0.018			
Sucrose (S)	0.007±0.002	-	1:1 G:S	0.749±0.016			
DA	2.085±0.038	0.015±0.0.005	1:1 G:DA	2.063±0.065	0.769±0.019	+171.8	+3.6
			10:1 G:DA	1.274±0.038	0.736±0.018	+67.9	-0.8
AA	0.835±0.012	0.022±0.0.008	1:1 G:AA	1.157±0.042	0.734±0.016	+52.4	-1.1
			10:1 G:AA	0.910±0.023	0.727±0.014	+19.9	-2.0
UA	1.103±0.039	0.033±0.0.009	1:1 G:UA	1.356±0.042	0.730±0.017	+78.7	-1.6
			10:1 G:UA	0.943 ±0.031	0.718±0,013	+24.2	-3.2
DA+AA+UA	2.121±0.065	0.161±0.011	10:1:1:1 G:DA:AA:UA	1.522 ±0.051	0.762±0.019	+100.5	+2.7

7. References

(1) Zhang, K.; Yang, W.; Liu, Y.; Zhang, K.; Chen, Y.; Yin, X. Laccase immobilized on chitosan-coated Fe_3O_4 nanoparticles as reusable biocatalyst for degradation of chlorophenol. *J. Mol. Struct.* **2020,** *1220*, No. 128769. DOI:10.1016/j.molstruc.2020.128769

(2) Ahangaran, F.; Hassanzadeh, A.; Nouri, S. Surface modification of Fe_3O_4@SiO_2 microsphere by silane coupling agent. *Int. Nano Lett.* **2013**, *3*, 3–7. DOI:10.1186/2228-5326-3-23

(3) Cui, Y.; Li, Y.; Yang, Y.; Liu, X.; Lei, L.; Zhou, L.; Pan, F. Facile synthesis of amino-silane modified superparamagnetic Fe_3O_4 nanoparticles and application for lipase immobilization. *J. Biotechnol.* **2010,** *150*, 171–174. DOI:10.1016/j.jbiotec.2010.07.013

(4) Chaichi, M.J.; Ehsani, M. A novel glucose sensor based on immobilization of glucose oxidase on the chitosan-coated Fe_3O_4 nanoparticles and the luminol-H_2O_2-gold nanoparticle chemiluminescence detection system. *Sens. Actuat. B Chem*. **2016**, *223*, 713–722.DOI: 10.1016/j.snb.2015.09.125

(5) Rossi, L. M.; Quach, A. D.; Rosenzweig, Z. Glucose oxidase-magnetite nanoparticle bioconjugate for glucose sensing. *Anal. Bioanal. Chem.* **2004,** 380, 606–613. DOI:10.1007/s00216-004-2770-3

(6) Liu, Y.; Fu, R.; Sun, Y.; Zhou, X.; Baig, S.A.; Xu, X. Multifunctional nanocomposites Fe_3O_4@SiO_2-EDTA for Pb(II) and Cu(II) removal from aqueous solutions. *Appl. Surf. Sci.* **2016,** *369*, 267–276. DOI:10.1016/j.apsusc.2016.02.043

(7) Villa, S.; Riani, P.; Locardi, F.; Canepa, F. Functionalization of Fe_3O_4 NPs by silanization: Use of amine (APTES) and thiol (MPTMS) silanes and their physical characterization. *Materials (Basel)* **2016**, *9,* No. 826. DOI:10.3390/ma9100826

(8) Shirali, M.; Mirhashemi, F. Epoxidation of vinyl cyanides by lithium hypochlorite in the presence of Fe_3O_4@Ag-CTAB as a new eco-friendly catalyst in aqueous medium. *Res. Chem. Intermed.* **2022,** *48*, 4063–4077. DOI:10.1007/s11164-022-04806-6

(9) Zdarta, J.; Bachosz, K.; Degórska, O.; Zdarta, A.; Kaczorek, E.; Pinelo, M.; Meyer, A.S.; Jesionowski, T. Co-immobilization of glucose dehydrogenase and xylose dehydrogenase as a new approach for simultaneous production of gluconic and xylonic acid. *Materials (Basel)* **2019**, *12*, 1–16. DOI:10.3390/ma12193167

(10) Moreira, K. da S.; de Oliveira, A. L. B.; Júnior, L.S. de M.; Monteiro1, R. R.C.; da Rocha1, T. N.; Menezes, L. F.; Fechine, L. M. U. D.; Denardin, J. C.; Michea, S.; Freire, R. M.; Fechine, P. B. A.; Souza M. C. M.; dos Santos, J. C. S. Lipase from rhizomucor miehei immobilized on magnetic nanoparticles: performance in fatty acid ethyl ester (FAEE) optimized production by the Taguchi method. *Fron. Bioeng. Biotechnol.* **2020,** *8*, 1-17 DOI:10.3389/fbioe.2020.00693

(11) Abboud, M.; Youssef S.; Podlecki, J.; Habchi, R.; Germanos, G.; Foucaran, A. Superparamagnetic Fe_3O_4 nanoparticles, synthesis and surface modification. *Mat. Sci. Semicon. Proc.* **2015***, 39*, 641–648 DOI: 10.1016/j.mssp.2015.05.035
